# C-Src confers resistance to mitotic stress through inhibition DMAP1/Bub3 complex formation in pancreatic cancer

**DOI:** 10.1186/s12943-018-0919-5

**Published:** 2018-12-15

**Authors:** Jingjie Li, Bin Hu, Ting Wang, Wenhua Huang, Chunmin Ma, Qin Zhao, Lingang Zhuo, Tao Zhang, Yuhui Jiang

**Affiliations:** 1 The Institute of Cell Metabolism, Shanghai Key Laboratory of Pancreatic Disease, Shanghai General Hospital, Shanghai Jiaotong University, School of Medicine, Shanghai, 200080 China; 2 Department of Gastroenterology, Shanghai General Hospital, Shanghai Jiao Tong University, School of Medicine, Shanghai, 200080 China; 3 Department of Pharmacology, College of Basic Medical Sciences, Tianjin Medical University, Tianjin, China; 4 Department of Orthopedics, Shanghai General Hospital, Shanghai Jiao Tong University, School of Medicine, Shanghai, 200080 China

**Keywords:** Mitosis,transcriptional reactivation,tumourigenesis,Bub3,p38,DMAP1,c-Src

## Abstract

**Background:**

Chromatin modification at mitosis is closely related to transcriptional reactivation in the subsequent cell cycle. We reasoned this process is deregulated by oncogenic signals, which would contribute to mitotic stress resistance in pancreatic cancer. Here, we show DMAP1/Bub3 complex mediates mitotic stress-induced cellular apoptosis, while this effect is counteracted by c-Src in pancreatic cancer cells. Our study aims to uncover an unidentified mechanism underlying the distinct response to mitotic stress between normal cells and pancreatic cancer cells.

**Methods:**

The interaction between Bub3 and DMAP1 upon mitotic stress signaling was determined through molecular and cell biological methods. The inhibitory effect of c-Src on DMAP1/Bub3-mediated DNA methylation and gene transcription profile was investigated. The association between c-Src-mediated DMAP1 phosphorylation and paclitaxel activity in vivo and clinicopathologic characteristics were analyzed.

**Results:**

Mitotic arrest induced p38-dependent phosphorylation of Bub3 at Ser211, which promotes DMAP1/Bub3 interaction. DMAP1/Bub3 complex is recruited by TAp73 to the promoter of anti-apoptotic gene *BCL2L1*, thus mediates the DNA methylation and represses gene transcription linked to cell apoptosis. Meanwhile, DMAP1 was highly phosphorylated at Tyr 246 by c-Src in pancreatic cancer cells, which impedes DMAP1/Bub3 interaction and the relevant cellular activites. Blocking DMAP1 pTyr-246 potentiates paclitaxel-inhibited tumor growth. Clinically, DMAP1 Tyr 246 phosphorylation correlates with c-Src activity in human pancreatic cancer specimens and poor prognosis in pancreatic cancer patients.

**Conclusions:**

Our findings reveal a regulatory role of Bub3 in DMAP1-mediated DNA methylation upon mitotic stress and provide the relevance of DMAP1 pTyr-246 to mitotic stress resistance during pancreatic cancer treatment.

**Electronic supplementary material:**

The online version of this article (10.1186/s12943-018-0919-5) contains supplementary material, which is available to authorized users.

## Introduction

During Mitosis, the duplicated chromosomes are separated into two daughter cells and its accuracy is critical for guaranteeing the integrity of genetic information [1]. The checkpoint responsible for the fidelity of chromosome segregation is termed spindle assembly checkpoint (also known as the mitotic checkpoint), which prevents chromosomes separation until each chromosome is correctly attached to the spindle apparatus. Mis-attachment of spindle to kinetchore leads to mitotic arrest resulted from mitotic checkpoint activation [2, 3]; and prolonged mitotic arrest accordingly initiates responsive signals that impede cell proliferation and survival, which avoids accumulation of cells with aneuploidy [4, 5]. Currently, the antimitotic agents such as paclitaxel, a microtubule-stabilizing drug which can result in mitotic arrest and apoptotic cell death, have been broadly utilized during the clinical cancer treatment [6]. Pancreatic ductal adenocarcinoma (PDAC) represents one of the most lethal diseases with approximately 6 months of median survival [7]. Up to now, the clinical regimens proven to increase survival for patients with advanced metastatic PDAC include standard gemcitabine and gemcitabine-based therapies [8]. Previous studies and clinical trials demonstrate that administration of paclitaxel in combination with gemcitabine shows a survival benefit over gemcitabine alone in advanced PDAC. However, a major conundrum for the clinical PDAC treatment is its innate resistance to available chemotherapies [9]. Clinical data indicates although effective clinical responses to antimitotic agents initially, a significant proportion of patients eventually develop resistance to the treatment [10]. This issue causes a large number of studies which aim to uncover mechanisms underlying the drug resistance, and multiple related cellular targets have been identified in a context dependent manner [11–15]. Further investigation in this regard would be important for a better understanding of how cancer cells escape unbeneficial effects from mitotic arrest and improving the relevant clinical therapy.

Epigenetic state of chromatin is closely related to mitosis progression [16–19]. Although transcription activity in mitosis is entirely silenced, the altered profile of epigenetic modifications at mitosis can exhibit their impacts on the transcriptional reactivation in the subsequent cell cycle [20–23]. Given this, it could be inferred that epigenetic regulation at mitosis under mitotic arrest would be involved in the modulation of genes transcription and relevant cellular activities postmitosis. In this regard, the distinct response to mitotic arrest between normal cell and cancer cell might accordingly produce different physiological outcomes.

Bub3 belongs to spindle assembly checkpoint (SAC) components which forms complex with Bub1 to mediate inhibitory effect on the ubiquitin ligase activity of the anaphase-promoting complex/cyclosome (APC/C) and the proteasome-mediated destruction of securin and cyclin B in mitosis [2, 24]. In contrast with the general mechanism, previous studies have revealed that the involvement of Bub3 in SAC can be regulated in a manner specific for cancer cells [25, 26]. Besides the canonical implication in SAC, Bub3 also potentially participates into other cellular activities including the regulation of DNA damage response and gene transcription [27–29]. In addition, Bub3 is found to interact with non-SAC protein, among which TAp73,a transcriptional factor, is well investigated and shown to importantly regulate SAC protein localization and activities [30, 31]. TAp73 belongs to p53 family and is characterized by its pro-apoptotic activities similar to those of p53, in contrast with the anti-apoptotic function of the ΔNp73 subfamily (lacking the TA domain) [32]. Thus, it would be interesting to study whether Bub3 is involved in the regulation of transcriptional activity of TAp73 and the relevant physiological function.

DMAP1 was initially identified as a protein associated with the N-terminal domain of DNA methytransferase 1 (DNMT1), the major enzyme that mediates mammalian DNA methylation [33, 34]. DMAP1/DNMT complex importantly contributes to transcriptional repression via the maintenance of methyl-CpG, in which a series of functional components such like transcriptional co-repressor TSG101, histone deacetylases (HDACs) are involved [35]. With respect to cell cycle progression, the effect of DMAP1 on transcriptional activity has been linked to G1/S and G2/M transition [36, 37]; while the regulation of DNA methylation by DMAP1 in mitosis and its potential to transcriptional reactivation postmitosis remains elusive.

Here, we found p38-dependent Bub3 phosphorylation promotes its binding to DMAP1 under mitotic arrest. Through interaction with TAp73, Bub3/DMAP1 potentiates DNMT1-mediated DNA methylation at the promoter region of anti-apoptotic genes, which blocks gene transcription post mitosis and leads to increased cellular apoptosis. In pancreatic cancer cells, DMAP1 is highly phosphorylated by c-Src; this phosphorylation abrogates DMAP1/Bub3 complex formation under mitotic arrest and eventually maintains cell survival.

## Materials and methods

### Antibodies

Antibodies that recognize DMAP1, Src, phosphor-Src pY416, CyclinB1, CyclinD1 was obtained from Cell Signaling Technology. Antibodies that recognize Flag, HA, actin and horseradish peroxidase (HRP)-conjugated secondary antibodies were purchased from Sigma. Antibody against Bub3 was obtained from Santa Cruz Biotechnology. Antibody against TAp73 antibody was from Millipore. Rabbit polyclonal Bub3 pSer-211 and DMAP1 pTyr-246 antibody was made by Signalway Antibody.

A peptide containing Bub3 pSer-211 or DMAP1 pTyr-246 was injected into rabbits. The rabbit serum was collected and purified using an affinity column with non-phosphorylated Bub3 Ser-211 or DMAP1 Tyr-246 peptides to exclude the antibodies for non-phosphorylated Bub3 or DMAP1, followed by an affinity column with phosphorylated Bub3 Ser-211 or DMAP1 Tyr-246 peptides to bind to and purify the Bub3 pSer-211 or DMAP1 pTyr-246 antibody. The Bub3 pSer-211 and DMAP1 pTyr-246 antibodies were then eluted and concentrated. Thymidine and Nocodazole were purchased from Sigma. Glutathione SepharoseTM 4B was obtained from GE Healthcare. FITC-AnnexinV apoptosis kit was from BD.

### Cell culture

Pancreatic cancer cell line PANC-1 was grown in Dulbecco’s modified Eagle’s medium (DMEM) supplemented with 10% FBS; SW1990 cells were maintained in PRMI-1640 plus 10% FBS. Immortal human pancreatic ductal epithelial cell line HPDE was cultured in DMEM supplemented with 10% FBS.

### DNA constructs and mutagenesis

The DNA constructs and mutagenesis were described as previously (41). pLKO.1 human DMAP1 shRNA was generated with the oligonucleotide CCGGATCAGCAAGAAGGACATTATCCTCGAGGATAATGTCCTTCTTGCTGATTTTTTG (Sigma). pLKO.1 human TAp73 shRNA was generated with the oligonucleotide CCGGCCAAGGGTTACAGAGCATTTACTCGAGTAAATGCTCTGTAACCCTTGGTTTTTG (Sigma). The pcDNA3.1-DMAP1-Flag was obtained from Changsha You Bao Biotechnology Co. Ltd. (Changsha, China). DNA sequences encoding Bub3 and DMAP1 were inserted into the pcDNA3.1 vector and Flag or HA tag was fused to the N terminus.

### Transfection

HPDE, PANC-1 and SW1990 cells were transfected with various plasmids using lipofectamine 2000 (Invitrogen) according to the vendor’s instructions.

### Cell synchronization

For mitotic arrest, cells were first synchronized by thymidine double block (2 mM) for indicated length of time. Nocodazole was then added (200 nM) for 16 h. Mitotic cells were then harvested by gentle pipetting.

### Immunoprecipitaion and immunoblotting

Immunoprecipitaion and immunoblotting were performed as described previously(41). Briefly, cells were lysed in lysis buffer (50 mM Tris-HCl pH 7.4, 150 mM NaCl, 1 mM EDTA, 1% Triton X-100, 1% sodium deoxycholate, and 0.1% SDS and cocktails of proteinase and phosphatase inhibitors). For exogenous immunoprecipitation, cell lysates were centrifuged to remove the cell debris and incubated in flag-M2 beads (Sigma) at 4 °C overnight. The beads were boiled after extensive washing. The proteins were eluted with SDS-PAGE sample buffer, resolved via SDS polyacrylamide gels and transferred onto polyvinylidene fluoride (PVDF) membranes. Membranes were blocked with 5% BSA, incubated with indicated specific primary antibodies and exposed to horseradish peroxidase (HRP)-conjugated secondary antibody (Sigma). The chemiluminescence signals emanating from the membranes were detected by ECL reagent. For endogenous immunoprecipitation, cells were lysed in lysis buffer and incubated with indicated antibodies at 4 °C overnight. The immunoprecipitates were incubated with protein A/G agarose beads (Santa Cruz) for 2–3 h followed by five washes.

### Mass spectrometric analysis

Bub3-associated proteins under mitotic arrest were analyzed by LC-MS/MS as described previously(41). Briefly, Flag-Bub3 associated proteins from the immune-precipitation assay were acetone-precipitated in vitro at − 20 °C overnight and resuspended in 50 mM ammonium bicarbonate buffer containing Rapigest (Waters Corp). The sample was heated to 95 °C for 10 min and allowed to cool down before 100 ng of sequencing-grade modified trypsin (Promega) was added. The digestion proceeded overnight at 37 °C and was analysed by LC-MS/MS using an Orbitrap-Elite mass spectrometer (Thermo Fisher Scientific). Proteins were identified by comparing the fragment spectra against those in the SwissProt protein database (EBI) using Mascot v.2.3 (Matrix Science) and Sequest v.1.20 via Proteome Discoverer v.1.3 (Thermo Fisher Scientific) software.

### Microarray profiling analysis

Total mRNA was purified from cells expressing wildtype DMAP1 and Y246F mutant DMAP1. Expression profiling analysis was performed using Affymetrix U133A microchips.

### Gene expression analysis

Total RNA was isolated from cells using RNAzol RT (Molecular Research Center) following the manufacturer’s instructions. We synthesized cDNA from 1 μg total RNA using iScript cDNA synthesis kit (Bio-Rad) and quantified mRNA levels by Real-time PCR using SYBR Green (Bio-Rad). We ran samples in technical triplicates and calculated relative mRNA levels normalized to actin mRNA levels in the same samples. Primers for HMGA2 were: 5’ACTTCAGCCCAGGGACAAC 3′ (forward) and 5’ CACAATAGCGAAAGTCCGAGG 3′ (reverse). Primers for BCL2L1were: 5’ CCAGAAGGGACTGAATCGGAG 3 ‘(forward) and 5’CGTCTCCAACAATCACCCAACAC 3′(reverse).

### Chromatin immunoprecipitation (ChIP) assay

ChIP assay was conducted using the Chromatin immunoprecipitation kit (Millipore) according to the manufacturer’s guidelines. In brief, cells were sonicated on ice. The supernatant was incubated with IgG or indicated antibodies at 4 °C. Genomic DNA in immune complexes was extracted and prepared for PCR reactions. Quantitative real-time PCR was used to measure the amount of bound DNA, and the value of enrichment was calculated according to the relative amount of input and the ratio to IgG. The primers covering TAp73 binding site of human BCL2L1 were: 5’CACGCCTGTAAACGATTCTCC3′ (forward) and 5’GCAGACTCGCTTACAGGAGAATC 3’ (reverse). Control primers selected against a specific region of chromosome 12 were 5’ATGGTTGCCACTGGGGATCT3’ (forward) and 5’TGCCAAAGCCTAGGGGAAGA3’(reverse).

### Pyrosequencing methylation analysis

Bisulphite treatment of 2 μg of genome DNA was undertaken using the EZ DNA Methylation Kit™ (Zymo Research). Hot-start PCR was carried out with HotStar Taq® Master Mix Kit (Qiagen Ltd.) using 3 μl bisulphite treated DNA. The targeted CpGs (BCL2L1 promoter CpG island) were selected and Pyrosequencing was carried out using the PyroMark Q96 ID (QIAGEN) system according to manufacturer’s protocol. The methylation status was automatically analyzed by Pyro Q-CpG software. Primers against the sequence included in BCL2L1 promoter region were:5’ATATATTGTGTGGTTTAGGGTGTAA3’(forward),5’biotin-AAAACCAACCTAACCAATATAATAAAACCC3’(reverse), and AGTAGTTGGGATTATAGG.

### Whole-genome bisulphate sequencing (WGBS) analysis

Genomic DNA from SW1990 cells expressing indicated plasmids was extracted using the Masterpure DNA Purification kit (Epicentre). For normal BS-seq library constructing, genomic DNA was fragmented to an average size of 350 bp using a Covaris S2 sonicator. Bisulfite sequencing libraries were constructed using the Illumina TruSeq DNA Library Preparation kit protocol. Following fragmentation, libraries were constructed using the Illumina Paired-End protocol consisting of end repair, “A” base addition and methylated-adaptor ligation. Ligated DNA was bisulfite converted using the EZ DNA Methylation-Gold kit (ZYMO). Final libraries were run on the 2100 Bioanalyzer (Agilent) using the High-Sensitivity DNA assay for quality control purposes. To achieve > 10× genome coverage, libraries were quan¬tified by qPCR with the Library Quantification kit for Illumina sequencing platforms (KK4824, Kapa Biosystems), using the 7900HT Real-Time PCR System (Applied Biosystems), and sequenced on the Illumina HiSeq 2000. The sequences alignment and methylation calling were performed with version 2.43 of BSMAP software.

### Recombinant protein purification

WT and mutant GST-Bub3, His-Bub3 and GST-DMAP1 were expressed in bacteria and purified, as described previously(41).

### In vitro kinase assay

Bacterially purified recombinant WT and mutant His-Bub3 was incubated with His-p38 in kinase assay buffer supplemented with 50 mM Tris-HCl, pH 7.5, 100 mM KCl, 50 mM MgCl_2_, 1 mM Na_3_VO_4_, 1 mM dithiothreitol (DTT), 5% glycerol, 0.2 mM ATP, 10 μCi of [γ − ^32^P]ATP) at 25 °C for 1 h. After the reaction, p38 was removed by extensive washing with RIPA buffer and kinase assay buffer. Purified recombinant WT and mutant GST-DMAP1 was incubated with His-c-Src in kinase assay buffer supplemented with 50 mM Tris-HCl, pH 7.5, 100 mM KCl, 50 mM MnCl2, 1 mM Na3VO4, 1 mM dithiothreitol (DTT), 5% glycerol, 0.2 mM ATP, 10 μCi of [γ − 32P]ATP) at 25 °C for 1 h.The His-Bub3 bound beads were recovered by centrifugation. For kinase phosphorylation analyses, the His-Bub3 bound beads were subjected to SDS-PAGE, and then autoradiography after incubation with EN3HANCE (PerkinElmer). The GST-Bub3 bound beads after p38 treatment in absence of hot ATP were further incubate with GST-DMAP1 for protein interaction assay as indicated.

### Apoptosis assay

Apoptosis assay was performed as instructed by the manufacturer (BD). Cells were resuspended in binding buffer containing FITC-conjugated Annexin V and PI. Cell apoptosis was detected by a flow cytometer (BD).

### Cell cycle analysis

Cells were fixed in 70% ice-cold ethanol at − 20 °C for 24 h. After several washes, fixed cells were resuspened in PBS containing RNase and PI solution and incubated at 37 °C for 30 min in darkness. Cell cycle distribution was analyzed by a flow cytometer (BD).

### Mouse

Six-week-old male nu/nu mice were subcutaneously injected with 5 × 10^6^ SW1990 cells in a volume of 150 μl of PBS/Matrigel (1/1 (*v*/v)). Primary tumour size was measured by a digital caliper. All animal experiments conformed to the guidelines of the Institutional Animal Care and Use Committee of Shanghai Jiaotong University.

### IHC

Ninety pairs of human PDAC tissues and their matched adjacent-tumour samples were collected from patients who had undergone a pancreatectomy at the Shanghai General Hospital. All samples used in this research were obtained with written informed consent from patients. The use of human tissues was approved by the Institutional Ethics Board of Shanghai General Hospital and conforms to the Helsinki Declaration and to local legislation. Immunohistochemistry was performed on paraffin-embedded sections of human pancreata (90 pancreatic cancer tissues). Human pancreatic cancer tissue sections (3–5 μm) were deparaffinized and rehydrated. Antigen retrieval was performed by boiling tissue sections in 10 mM citrate buffer (pH 6.0) in a microwave oven for 30 min. The activity of endogenous peroxidase was blocked with 3% hydrogen peroxide in methanol for 10 min at room temperature. After washing, non-specific binding sites were blocked by incubating the slides with 10% FBS/PBS for 30 min at room temperature. Sections were subsequently incubated with rabbit polyclonal anti-DMAP1 pTyr-246 (Signalway, 1:50) at 4 °C overnight. After incubation with the primary antibody, the sections were washed and incubated with secondary antibodies and DAB staining reagent with GTVisionTM Detection System/Mo&Rb Kit according to manufacturer’s instructions. After counterstain with hematoxylin and dehydration, the sections were mounted and imaged using the Leica microscope. Immunoreactivity was semi-quantitatively evaluated according to intensity and area: the staining intensity of pancreatic cancer cells themselves was recorded as “weak (0–2)” or “strong staining (2.1-5)”. Statistical analysis of the survival time for 90 patients with low vs moderate or high DMAP1 pTyr-246 level, was performed using the GraphPad Prism 5 Software. The Kaplan-Meier method and log-rank tests were used for survival analysis. Statistical significance was set at *p* < 0.05. The cox multivariate analysis with patient sex and age was performed with SPSS 19 Software.

### Statistical analysis

Statistical analysis was conducted with the two-tailed unpaired Student’s t-test. All data represent the mean ± s.e.m. of three independent experiments.

## Results

### Bub3 interacts with DMAP1 during mitotic arrest

**Fig. 1:**
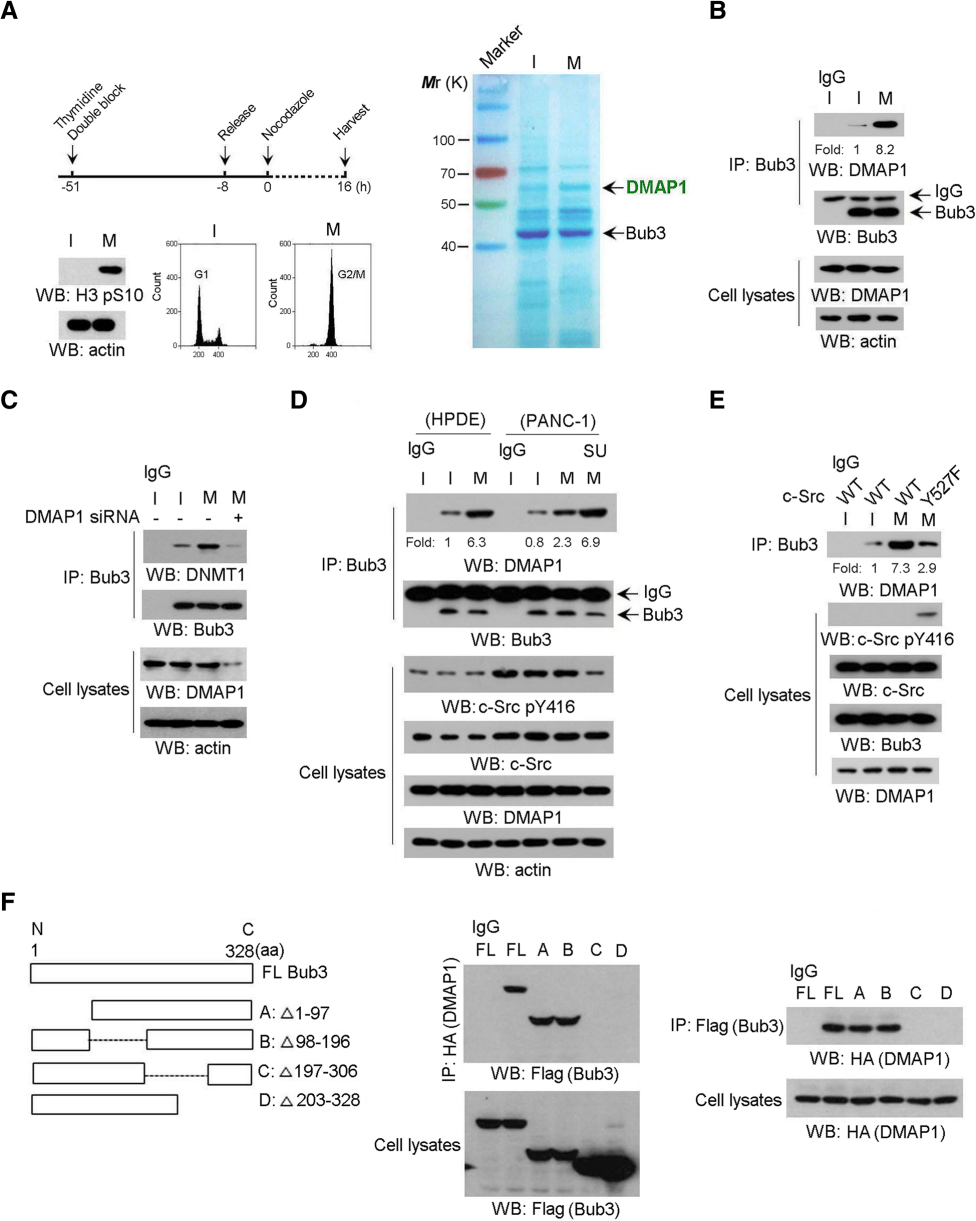
Bub3 interacts with DMAP1 during mitotic arrest. In A-F, immunoblotting analyses were performed using the indicated antibodies; Data represent 1 out of 3 experiments. **a**, HPDE cells expressing Flag-Bub3 were synchronized in interphase by thymidine double block (2 mM) or were synchronized in mitosis by nocodazole (200 nM) treatment for 16 h after releasing thymidine double block for 8 h (left panel), then cells were extracted and subjected to immunoprecipitation with an anti-Flag antibody. The precipitates from immunoprecipitated-Flag-bub3 were washed by Flag peptides and then were analyzed by coomassie brilliant blue staining and immunoblotting (right panel). **b**, HPDE cells were synchronized by thymidine double block (2 mM) and were released for 8 h, followed by nocodazole (200 nM) treatment for 16 h, cellular extracts were subjected to immunoprecipitation with an anti-Bub3 antibody. **c**, HPDE cells were transfected with or without DMAP1 siRNA. Cellular extracts were subjected to immunoprecipitation with an anti-Bub3 antibody. **d**, HPDE and PANC-1 cells were synchronized by thymidine double block (2 mM) and were released for 8 h, followed by nocodazole (200 nM) treatment for 16 h (left panel). PANC-1 cells were treated with SU6656 (shown as ‘SU’) (10 μM) for 1 h post Nocodazole treatment for 16 h. Cellular extracts were subjected to immunoprecipitation with an anti-Bub3 antibody. **e**, HPDE cells overexpressed with Src Y527F were synchronized by thymidine double block (2 mM) and were released for 8 h, followed by nocodazole (200 nM) treatment for 16 h (left panel). Cellular extracts subjected to immunoprecipitation with an anti-Bub3 antibody. **f**, HPDE cells synchronized by thymidine double block (2 mM) were released for 8 h, followed by nocodazole (200 nM) treatment for 16 h (left panel). Cells expressing HA-DMAP1was transfected with plasmids expressing indicated length of Flag-Bub3. Cellular extracts were subjected to immunoprecipitation with an anti-Flag antibody

To determine the potential effects of Bub3 on cellular events in addition to its canonical function relevant to spindle checkpoint, human normal pancreatic duct epithelial HPDE cells were stably expressed with Flag-Bub3 and were arrested in mitosis by Nocodazole treatment following synchronization with thymidine double block. The efficiency of mitotic arrest was validated by flow cytometry analyses and examination of histone H3 Ser 10 phosphorylation, a marker of late G2 phase and mitosis (Fig. 1a, the left panel). Immunoprecipitation with an anti-Flag antibody followed by mass spectrometry (Fig. 1a, the right panel, and Additional file 1: Figure S1A) indicated mitotic arrest dramatically promoted the interaction between Bub3 and DNA methytransferase 1 (DNMT1) associated protein DMAP1. Accordingly, the binding of DNMT1 to Bub3 was also increased at this condition, although to a less extent compared with DMAP1 (Additional file 1: Figure S1B). In line with previous finding [30], TAp73 was detected in Flag-Bub3 precipitates at mitosis (Additional file 1: Figure S1C). The interaction between endogenous Bub3 and DMAP1 under mitotic arrest was further verified through reciprocal co-immunoprecipitation analyses by using antibodies against Bub3 (Fig. 1b and Additional file 1: Figure S1D) and DMAP1 (Additional file 1: Figure S1E) respectively. In addition, co-immunoprecipitation analyses further showed that DNMT1 depletion had no effect on the interaction between DMAP1 and Bub3 (Additional file 1: Figure S1F); however, DMAP1 depletion led to a notable decrease of Bub3/DNMT1 complexes formation (Fig. 1c), suggesting DMAP1 mediates Bub3-DNMT1 complex formation under mitotic arrest.

Subsequently, Bub3/DMAP1 interaction was examined in pancreatic cancer cell PANC-1 cells and SW1990 cells (Additional file 1: Figure S1G). Unexpectedly, mitotic arrest only led to a slight increase of DMAP1/Bub3 interaction in cancer cells (Fig. 1d), implying certain cancer-specific signaling participate in the regulation of Bub3/DMAP1 complex formation. The hyperactive c-Src signaling has been implicated in pancreatic cancer development [38, 39]. PANC-1 and SW1990 cells were treated with c-Src inhibitor SU6656 that did not show significant effect on mitotic index (Additional file 1: Figure S1H). As a result, c-Src inhibition (the reduced Y416 phosphorylation level) evidently enhanced induced Bub3/DMAP1 complex formation (Fig. 1d) under mitotic arrest, suggesting c-Src plays a negative role in Bub3/DMAP1 interaction. To support this finding, we expressed a constitutively active c-Src (c-Src Y527F) in HPDE cells and found c-Src Y527F largely blocked mitotic arrest-induced DMAP1/Bub3 interaction (Fig. 1e). To further characterize DMAP1-binding region of Bub3, we constructed series truncates of Bub3 spanning various amino acids sequence (Fig. 1f). Co-immunoprecipitation analyses in mitotic-arrested HPDE cells indicated the Bub3 truncates with deletion of 197-306aa (amino acid) or 203-328aa instead of 1-97aa or 98-196aa lost the ability of DMAP1binding, revealing DMAP1-binding region of Bub3 is located in the amino acid sequence between 203aa and 306aa (Fig. 1f, right panel).

### p38 phosphorylates Bub3 at Ser211 and promotes Bub3/DMAP1interaction

**Fig. 2:**
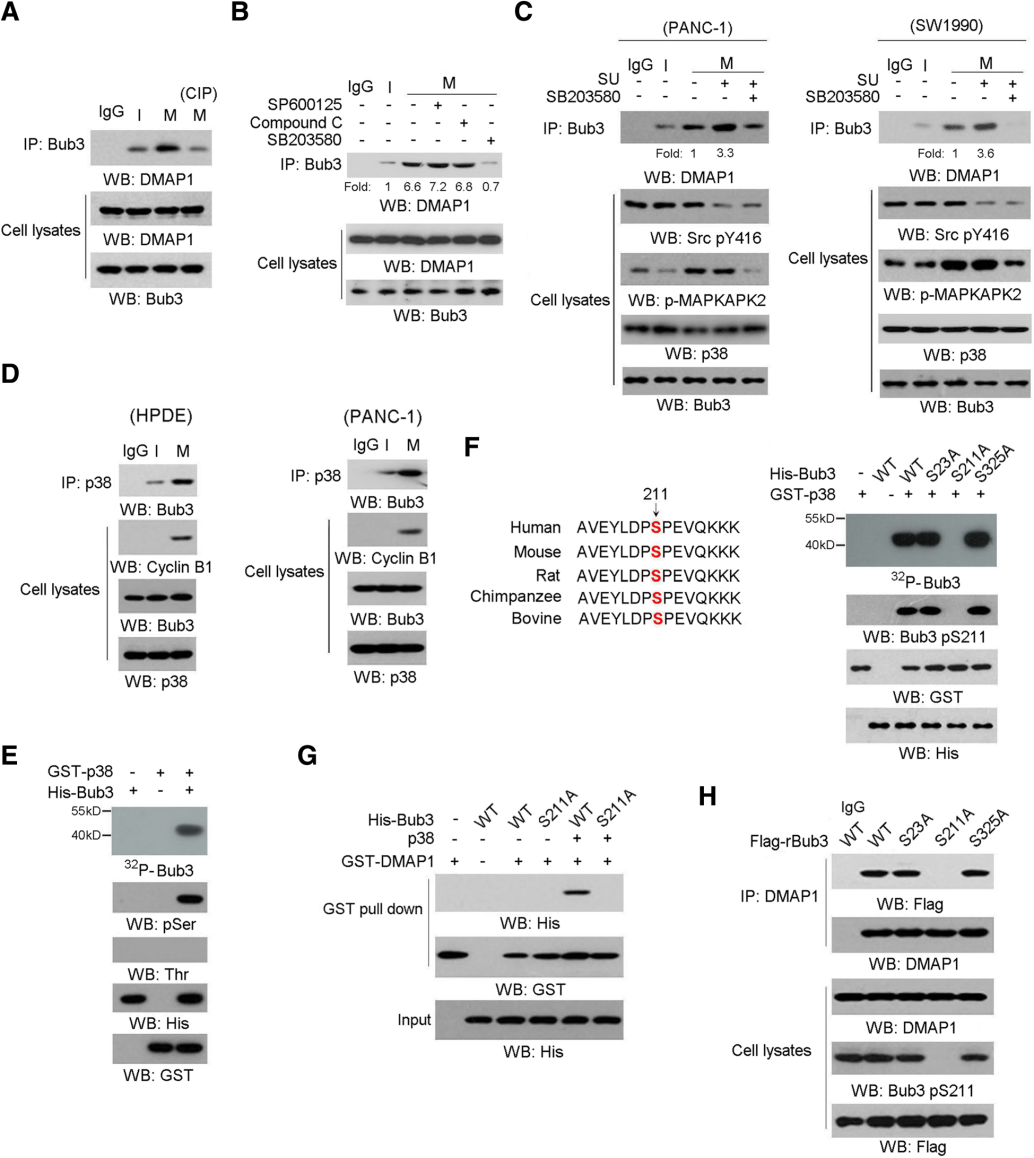
p38 phosphorylates Bub3 at Ser211 and promotes Bub3/DMAP1interaction. In **a**-**h**, immunoblotting analyses were performed using the indicated antibodies. Data represent 1 out of 3 experiments. In **a**-**d** and **h**, cells were synchronized in interphase (I) by thymidine (2 mM) double block or were synchronized in mitosis (M) by nocodazole (200 nM) treatment for 16 h after releasing thymidine double block for 8 h. In **a**-**h**, data represent 1 out of 3 experiments. **a**, HPDE cells were synchronized in interphase or mitosis. Cellular extracts were subjected to immunoprecipitation with an anti-Bub3 antibody and the immunoprecipitates were treated with or without CIP (10 units). **b**, HPDE cells synchronized in interphase or mitosis were treated with Compound C (10 μM), SP600125 (20 μM) and SB203580 (25 μM) for 1 h, after Nocodazole treatment for 16 h. **c**, PANC-1 or SW1990 cells were synchronized in interphase or mitosis. Cells were treated with SB203580 (25 μM) and SU6656 (shown as ‘SU’) (10 μM) for 1 h, after Nocodazole treatment for 16 h. Cellular extracts were subjected to immunoprecipitation with an anti-Bub3 antibody. **d**, HPDE or PANC-1 cells were synchronized in interphase or mitosis. Cellular extracts were subjected to immunoprecipitation with an anti-p38 antibody. **e** and **f**, In vitro phosphorylation analyses were performed by mixing the purified active p38 with the indicated purified GST-Bub3 proteins in the presence of [γ-32P]ATP. Ser211 of Bub3 is evolutionarily conserved in the indicated species (**f**, left panel). **g**, The indicated purified His-Bub3 protein was mixed with GST-DMAP1 purified protein with or without p38. GST pull down analyses were performed. **h**, HPDE cells synchronized in mitosis were expressed with indicated Flag-Bub3. Cellular extracts were subjected to immunoprecipitation with an anti-Flag antibody

We set forth to investigate the upstream signals that mediate Bub3/DMAP1 complex formation. Interestingly, the interaction between DMAP1 and Bub3 was disrupted by treatment of calf intestinal phosphatase (CIP) but not Na_3_VO_4_ in precipitates from Bub3 (Fig. 2a and Additional file 2: Figure S2A), indicating Bub3/DMAP1 interaction is phosphorylation dependent. Several Ser/Thr protein kinases, including c-Jun N-terminal kinase JNK, Adenosine 5′-monophosphate (AMP)-activated protein kinase AMPK, and p38 mitogen-activated protein kinases p38 are importantly involved in cellular stress signaling [40], of which the responses to mitotic arrest were detected (Additional file 2: Figure S2B). HPDE cells were pre-treated with SP600125 (JNK inhibitor), Compound C (AMPK inhibitor) and SB203580 (p38 inhibitor) and the according efficiency was examined (Additional file 2: Figure S2C). Notably, mitotic arrest-induced Bub3/DMAP1 interaction was disrupted specifically by SB203580 treatment (Fig. 2b). In agreement, pre-treatment with SB203580 largely blocked mitotic arrest-induced Bub3/DMAP1 interaction in PANC-1 cells and SW1990 cells with c-Src inhibition (Fig. 2c). These results indicated p38 activity was required for Bub3/DMAP1 interaction. In addition, co-immunoprecipitation analyses indicated mitotic arrest promoted the binding of p38 to Bub3 either in HPDE cells (Fig. 2d, left panel) or in PANC-1 cells (Fig. 2 d, right panel); this forced us to further investigate whether Bub3 is the substrate of p38 kinase. An in vitro protein phosphorylation assay showed that purified, activated p38 phosphorylated purified recombinant Bub3 and the phosphorylation was detected only with an anti-phospho-Serine antibody (Fig. 2e). Scansite analysis predicted a couple of potential p38-phosphorylated residues in the amino acid sequence of Bub3 (Fig. 2f, the left panel), and further in vitro protein phosphorylation assay showed that only mutation of evolutionarily conserved Ser 211 into alanine abolished p38-mediated Bub3 phosphorylation, as demonstrated by autoradiography and immunoblotting analyses using a specific Bub3 pSer-211 antibody (Fig. 2f, the right panel). Accordingly, mitotic arrest-induced Bub3 Ser-211 phosphorylation was abolished by p38 depletion or SB203580 (Additional file 2: Figure S2D and S2E) but not CDK1 inhibitor RO3306 treatment (Additional file 2: Figure S2F). To investigate whether Bub3 pS211 is addicted to p38 activity in mitosis, we synchronized HPDE cells in G1 phase and treated cells with TGF-β, which resulted in p38 activation. As shown in Additional file 2: Figure S2G, neither p38/Bub3 interaction nor Bub3 S211 phosphorylation was changed by TGF-β. These data suggest the high availability of p38 activity for Bub3 in mitotic arrest is critical for Bub3 S211 phophorylation. To evaluate the effects of Bub3 S211 phophorylation on the interaction between Bub3 and DMAP1, we performed a GST pull assay in which recombinant GST-DMAP1 were mixed with immobilized recombinant WT His-Bub3 or His-Bub3 S211A on agarose beads, with or without addition of purified p38. Interestingly, we found WT Bub3 but not Bub3 S211A mutant is able to interact with DMAP1 in presence of p38 (Fig. 2g). Consistently, another GST pull assay showed the phosphor-mimic mutant His-Bub3 S211D was capable of binding to GST-DMAP1 in the absence of p38 (Additional file 2: Figure S2H). These results suggest p38-mediated Bub3 S211 phosphorylation is sufficient for its binding to DMAP1. Subsequently, endogenous Bub3 was depleted and expression of RNAi-resistant WT rBub3 or rBub3 mutants were reconstituted in HPDE cells (Additional file 2: Figure S2I). Co-immunoprecipitation analyses indicated rBub3 S211A but not WT rBub3 failed to interact with DMAP1 and undergo Bub3 S211 phophorylation under mitotic arrest in HPDE cells (Fig. 2h). In accordance, mitotic arrest notably induced the binding of DMAP1 to WT-Bub3 but not Bub3 S211A in PANC-1 cells with Src inhibition (Additional file 2: Figure S2J). Of note, Bub3 S211A was still able to form complex with the spindle checkpoint protein, Bub1 and BubR1 (Additional file 2: Figure S2K). These results suggest p38 mediated Bub3 S211 phosphorylation is required for its binding to DMAP1 under mitotic stress.

### C-Src phosphorylates DMAP1 at Tyr246 and disrupts Bub3/DMAP1 complex formation

**Fig. 3:**
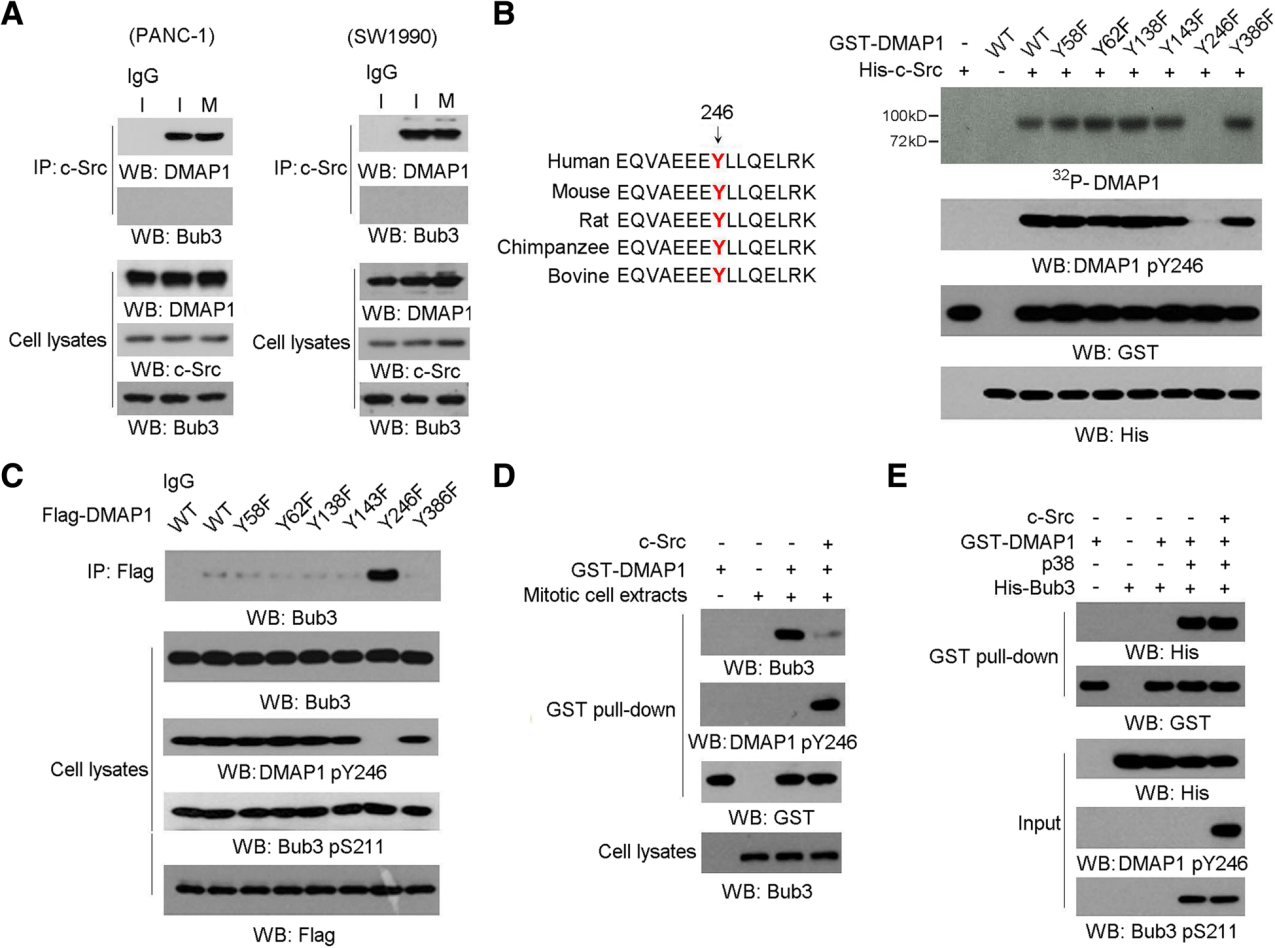
c-Src phosphorylates DMAP1 at Tyr246 and disrupts Bub3/DMAP1 complex formation. In **a**-**e**, immunoblotting analyses were performed using the indicated antibodies. Data represent 1 out of 3 experiments. In **a**, **c** and **d**, cells were synchronized in interphase (I) by thymidine double block (2 mM) or were synchronized in mitosis (M) by nocodazole (200 nM) treatment for 16 h after releasing thymidine double block for 8 h. In **a**-**e**, data represent 1 out of 3 experiments. **a**, PANC-1 or SW1990 cells were synchronized in interphase or mitosis. Cellular extracts were subjected to immunoprecipitation with an anti-Src antibody. **b**, In vitro phosphorylation analyses were performed by mixing the purified active c-Src with the indicated purified GST-DMAP1 proteins in the presence of [γ-^32^P]ATP. Tyr246 of DMAP1 is evolutionarily conserved in the indicated species (**b**, left panel). **c**, Cells synchronized in mitosis were expressed with indicated Flag-DMAP1. Cellular extracts were subjected to immunoprecipitation with an anti-Flag antibody Cells. **d**, The indicated purified GST-DMAP1 protein was mixed with mitotic extracts from PANC-1 cells in the presence or absence of c-Src. GST pull down analyses were performed. **e**, Purified His-Bub3 protein with or without phosphorylation by c-Src was mixed with GST-DMAP1 purified protein with or without p38. GST pull down analyses were performed

Mitotic arrest led to a dramatic increase of Bub3 S211 phosphorylation in PANC-1 cells regardless of c-Src activity status (Additional file 2: Figure S2J); this suggests c-Src negatively regulates Bub3/DMAP1 interaction in a manner independent on Bub3 pS211 levels. Co-immunoprecipitation analyses in PANC-1 or SW1990 cells showed c-Src was constantly associated with DMAP1 but not Bub3 throughout the cell cycle (Fig. 3a), which raises the possibility that DMAP1 is a substrate of c-Src kinase. According to Scansite analysis, we found a number of potential Src-phosphorylated sites on DMAP1 amino acids sequence (Fig. 3b, the left panel) and that mutation of evolutionarily conserved Tyr 246 into phenylalanine largely abolished Src-mediated DMAP1 phosphorylation, as validated by autoradiography and immunoblotting analysis with a specific antibody against DMAP1 pY246 (Fig. 3b, the right panel). Immunoblotting analysis in PANC-1 cells showed a constant level of DMAP1 pY246 throughout the cell cycle, which were abolished by c-Src inhibition (Additional file 3: Figure S3A). Co-immunoprecipitation analyses showed either WT DMAP1 or DMAP1 Y246F mutant but not Bub3 were able to interact with c-Src (Additional file 3: Figure S3B). However, DMAP1 Y246F but not WT DMAP1 exhibited a strong ability of Bub3 binding in PANC1 cells under mitotic arrest (Fig. 3c), which indicated c-Src-mediated DMAP1 phosphorylation impeded Bub3/DMAP1 interaction. Of note, mitotic arrest-induced Bub3 S211 phosphorylation was not affected by expression of DMAP1 Y246F in PANC1 cells (Fig. 3c), which is in line with the results shown in Additional file 2: Figure S2J. Furthermore, GST pull down analysis revealed purified DMAP1 was substantially associated with Bub3 from cellular extract under mitotic arrest and this interaction was significantly compromised by addition of c-Src (Fig. 3d). In contrast, another GST pull down assay showed purified DMAP1 protein was still able to interact with Ser211-phosphorylated purified Bub3 in presence of c-Src, in which p38-mediated Bub3 S211 phosphorylation was not affected (Fig. 3e). These data imply the DMAP1 phosphorylation by c-Src does not directly interrupt Bub3/DMAP1 interaction and there is another unidentified component such like SH2 domain containing molecules, that can compete with Bub3 to interact with tyrosine phosphorylated DMAP1 under mitotic arrest.

### C-Src phosphorylates DMAP1 and blocks Bub3/DMAP1-repressed anti-apoptotic genes transcription

**Fig. 4:**
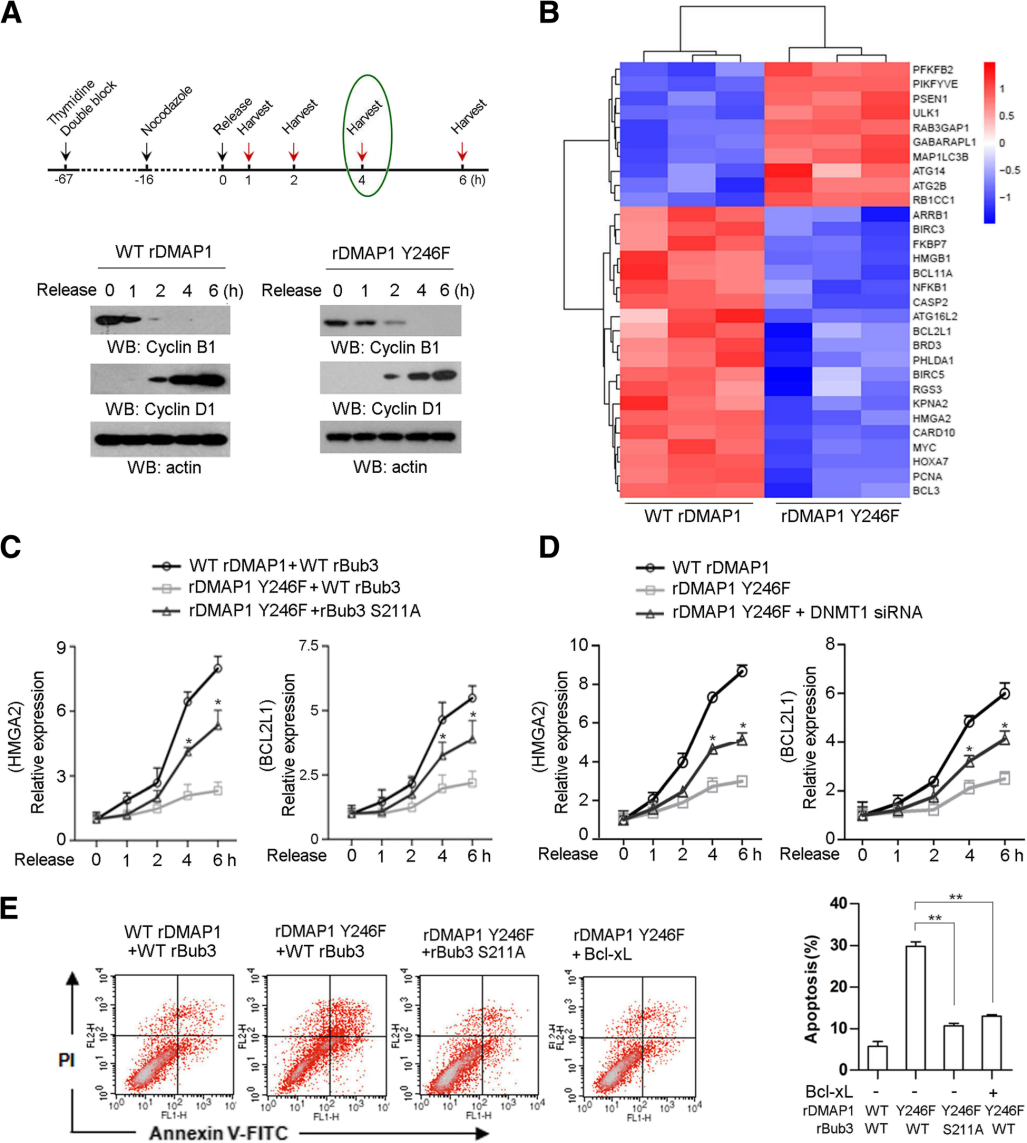
Bub3/DMAP1 complex represses anti-apoptotic genes transcription. In **a**, immunoblotting analyses were performed using the indicated antibodies; data represent 1 out of 3 experiments. In **c**-**e**, the values represent mean ± s.e.m. of three independent experiments. **a**, SW1990 cells were double blocked by thymide and treated with nocodazole (200 nM) following by releasing for the indicated periods. **b**, SW1990 cells were released for 4 h after thymidine double block and nocodazole (200 nM) for 16 h. Hierachical clustering of 4307 probe sets correlating with DMAP1 Y246F-expressed cells show that genes relevant to anti-apoptosis or autophagy were effective in separating cases from DMAP1 WT-expressed cells. **c** and **d** SW1990 cells expressed with the indicated plasmids were treated with nocodazole (200 nM) post thymidine double block, and were released for the indicated time. Relative mRNA levels were analyzed by real-time PCR. In **c**, * represents *p* < 0.05 between groups of cells expressing rDMAP1 Y246F plus WT rBub3 and groups of cells expressing rDMAP1 Y246F plus rBub3 S211A. In **d**, * represents *p* < 0.05 between groups of cells expressing rDMAP1 Y246F and groups of cells expressing rDMAP1 Y246F plus DNMT1 siRNA. **e**, Cell apoptosis was analyzed by Annexin V assays followed by flow cytometry. ** represents *p* < 0.01 between indicated groups

DMAP1 participates in transcriptional repression in complex with DNMT1 [33]. To investigate whether the inhibitory effect of c-Src-mediated DMAP1 phosphorylation on Bub3/DMAP1 interaction is involved in the regulation of postmitotic gene transcription, we depleted endogenous DMAP1 in SW1990 cells and reconstituted the expression of RNAi-resistant WT rDMAP1 or DMAP1 rY246F (Additional file 4: Figure S4A), and analysis of cell synchronization indicated cell cycle progression was unchanged between cells expressing WT rDMAP1 and rY246F DMAP1 (Fig. 4a and Additional file 4: Figure S4B). Subsequently, the global gene expression profiles were examined by cDNA microarray analysis and gene expression between cells expressing WT rDMAP1 and rY246F DMAP1 were compared. We noted a number of transcripts that display lower levels in DMAP1 rY246F-expressing cells were enriched for genes implicated in anti-apoptosis, inflammation and autophagy, whereas transcripts showing high levels in DMAP1 rY246F-expressing cells were associated with pro-apoptosis or survival maintenance (Fig. 4b). Given the suppressive role of DMAP1 in transcription, we supposed genes that were downregulated by DMAP1 Y246F expression are potential targets of Bub3/DMAP1 complex. *HMGA2*, *BCL2L1*, *BRIC3*, *BRIC5*, and *NFKB1*, which are noted to encode critical anti-apoptotic proteins, were further selected to evaluate the effect of Bub3/DMAP1 complex on postmitotic gene reactivation. In line with the results from cDNA microarray analysis, real-time PCR analysis indicated that the reactivation of these genes were significantly delayed by expression of DMAP1 Y246F mutant especially for cells that exit mitosis for 4 h and 6 h (high proportion of cells in G1 phase) (Fig. 4c). Given the importance of Bub3 S211 phosphorylation in Bub3/DMAP1 interaction (Fig. 2h), its potential effect on transcription was investigated accordingly. SW1990 cells with Bub3 depletion were reconstituted the expression of RNAi-resistant WT rBub3 or rBub3 S211A (Additional file 4: Figure S4C). Apparently, expression of Bub3 S211A dramatically reversed the DMAP1 Y246F-repressed gene transcription (Fig. 4c and Additional file 4: Figure S4D). Meanwhile, the inhibitory effect of DMAP1 Y246F on gene transcription was found to be abrogated by DNMT1 depletion (Fig. 4d and Additional file 1: Figure S4E), implying DNA DNMT1/DNMT1 would be required for transcriptional regulation here. The suppressive effect of DMAP1 Y246F on postmitotic transcription prompts us to further examine its impact on cell survival under mitotic stress. As a result, Annexin V assays indicated expression of DMAP1 Y246F mutant led to an increased proportion of SW1990 cells undergoing apoptosis after mitosis exit; while this effect was most significantly attenuated by expression of Bub3 S211A or ectopic expression of *BCL2L1*-encoded Bcl-xL (Fig. 4e) instead of ectopic expression of *HMGA2*-encoded HMGA2, *BRIC5*-encoded cIAP-2, or *BRIC3*-endoced Survivin alone (Additional file 4: Figure S4F), revealing the critical role of *BCL2L1* transcription on mitotic stress-induced cell survival, which is inversely regulated by DMAP1 pY246 and Bub3 pS211. Above all, these results suggest Bub3/DMAP1 complex act as a repressive modulator of transcription for anti-apoptotic genes under mitotic stress and its effect is impaired in tumour cells with high levels of DMAP1 pY246.

### C-Src-mediated DMAP1 phosphorylation blocks Bub3/DMAP1-mediated DNA methylation

**Fig. 5:**
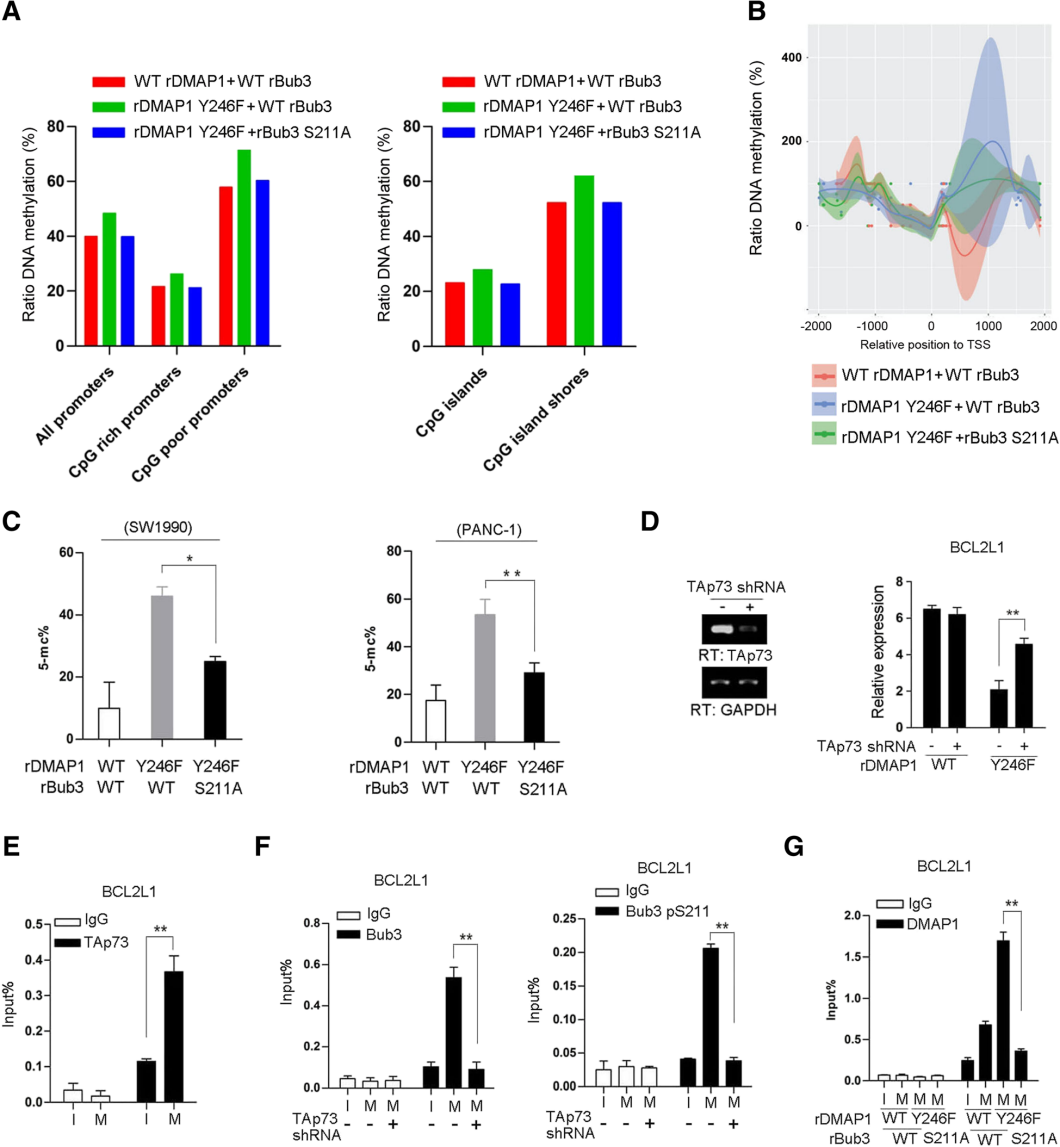
c-Src-mediated DMAP1 phosphorylation blocks DMAP1-mediated DNA methylation. **a**, SW1990 cells expressed with the indicated plasmids were synchronized in mitosis (M) by nocodazole (200 nM) treatment for 16 h after releasing thymidine double block for 8 h. DNA methylation levels of promoters and CpG islands or CpG islands shores were presented as ratio of methylated reads to unmethylated reads. The values represent from 2 repeated samples. **b**, SW1990 cells expressed with the indicated plasmids were synchronized in mitosis (M) by nocodazole (200 nM) treatment. DNA methylation profile of the promoter region (TSS ±1 kb) of *BCL2L1*. The values represent from 2 repeated samples. **c**, SW1990 and PANC-1 cells expressed with the indicated plasmids were synchronized in mitosis (M) by nocodazole (200 nM) treatment. 5-mc levels were examined by bisulfite sequencing using primers covering TAp73 binding site of BCL2L1 gene promoter region. **d**-**g**, Cells were synchronized in interphase (I) by thymidine (2 mM) double block or were synchronized in mitosis (M) by nocodazole (200 nM) treatment. **d**, SW1990 cells with depleted DMAP1, and reconstituted expression of WT rDMAP1 or rDMAP1 Y246F were transfected with plasmid for expression of TAp73 shRNA. Relative mRNA level was analyzed by real-time PCR. **e**, ChIP analyses were performed in SW1990 cells. The primers covering TAp73 binding site of *BCL2L1* gene promoter region were used for the real-time PCR. **f**, SW1990 cells were transfected with plasmid for expression of TAp73 shRNA. ChIP analyses were performed. The primers covering TAp73 binding site of *BCL2L1* gene promoter region were used for the real-time PCR. **g**, SW1990 cells were expressed with the indicated plasmids. ChIP analyses were performed. The primers covering TAp73 binding site of *BCL2L1* gene promoter region were used for the real-time PCR. The y axis shows the value normalized to the input. The values represent mean ± s.e.m. of three independent experiments;*represents *p* < 0.05 and **represents *p* < 0.01 between indicated groups

DMAP1/DNMT1 is known responsible for mammalian DNA methylation [33, 34]. We wondered the repressive role of DMAP1 pY246 in gene transcription was linked to its potential effect on DNA methylation. To this end, we performed whole-genome bisulphate sequencing (WGBS) analysis of SW1990 cells with reconstituted expression of WT rDMAP1, DMAP1 rY246F or DMAP1 rY246F plus rBub3 S211A (Additional file 4: Figure S4C), which displays an unbiased insight of changes in DNA methylation occurring genome-wide. After trimming adapters and filtering low-quality reads, we generated approximately 1.3 billion paired-end reads for each group, of which 82.3, 80 and 82.3% mapped uniquely to the reference genome respectively (Additional file 5: Figure S5A). We identified methylated cytosines using bismark methylation extractor software and binomial tests. Low methylated cytosines were all filtered out, and ca. 76 to 88 million methylated-cytosines (mCs) in each sample were identified. Compared with WT counterpart, we found mCs in the CG- but not CHG- or CHH-context rose dramatically in SW1990 cells with DMAP1 rY246F expression (Additional file 5: Figure S5B); while this effect was largely inhibited in cells with simultaneous expression of rBub3 S211A with DMAP1 rY246F expression. Similarly, screening for all autosomes indicated expression of DMAP1 rY246F but not rBub3 S211A plus DMAP1 rY246F led to a general increase of chromosome-associated methylation (Additional file 5: Figure S5C), and this increase was abundant along all genomic features with increases detected in promoters, exons, introns, intragenic regions (Additional file 5: Figure S5D). Meanwhile, analysis from a regulatory standpoint revealed DNA methylation at CpG rich and CpG poor promoters was both enhanced by DMAP1 rY246F expression (Fig. 5a, left panel). Accordingly, the cell group with DMAP1 rY246F expression resulted in a pattern of hypermethylation at the CpG rich islands as well as CpG island shores, compared with the other groups (Fig. 5a, right panel). Specifically, we further select *BCL2L1* to analyze the relevant gene DNA methylation density from WGBS data. All of the identified mCs were mapped to promoter upstream (− 1 kb) and downstream (+ 1 kb). As a result, the notable elevation of CG methylation was detected at promoter downstream region in SW1990 cells with expression of rDMAP1Y246F compared to WT rDMAP1, which was significantly reversed by concomitant expression of rBub3 S211A (Fig. 5b). Consistently, this observation was further confirmed by the additional methylation analysis in SW1990 cells (Fig. 5c, left panel and Additional file 5: Figure S5E, left panel) and well recapitulated in PANC-1 cells (Additional file 5: Figure S5E, right panel). Collectively, these results indicated DMAP1 pY246 plays a negative role in global DNA methylation of genome, and DMAP1-Bub3 complex formation is required for DNA methylation of specific genes.

Bub3 was associated with the transcriptional factor TAp73 during mitotic arrest (Additional file 1: Figure S1C), and the co-immunoprecipitation analysis showed this interaction was independent on Bub3 S211 phosphorylation (Additional file 5: Figure S5F). We wondered whether TAp73 was involved in the transcriptional regulation by Bub3/DMAP1 complex. As shown in Fig. 5d, TAp73 depletion reversed *BCL2L1* transcription in SW1990 cells expressed with DMAP1 Y246F, suggesting TAp73 is critical for transcription suppression mediated by DMAP1/Bub3. Sequence analysis revealed ‘ggcatgcgccaccacgcc’ at *BCL2L1* promoter are putative TAp73 binding sites and ChIP analyses indicated TAp73 was enriched at the promoter region covering the binding sites at mitosis (Fig. 5e). Additionally, *BCL2L1* promoter-associated Bub3 (Fig. 5f, left panel and Additional file 5: Figure S5G), Bub3 S211 phosphorylation (Fig. 5f, right panel) were also found to be significantly increased under mitotic arrest in SW1990 cells, which were blocked by TAp73 depletion. Similarly, mitotic arrest dramatically promoted the accumulation of DMAP1 (Fig. 5g) as well as DMNT1 (Additional file 5: Figure S5H) at *BCL2L1* promoter regions in SW1990 cells expressed with DMAP1 Y246F but not DMAP1 Y246F/Bub3 S211A, in comparison with the WT counterparts. In contrast, DMAP1 Y246 phosphorylation was failed to be detected at promoter sites during mitotic arrest (Additional file 5: Figure S5I). These data suggest TAp73 is responsible for the accumulation of Bub3 and DMAP1 complex at promoter, in which Bub3 S211 phosphorylation is indispensable for DMAP1/DNMT1 recruitment.

### DMAP1 Y246 phosphorylation counteracts antitumor activity of paclitaxel in vivo

**Fig. 6:**
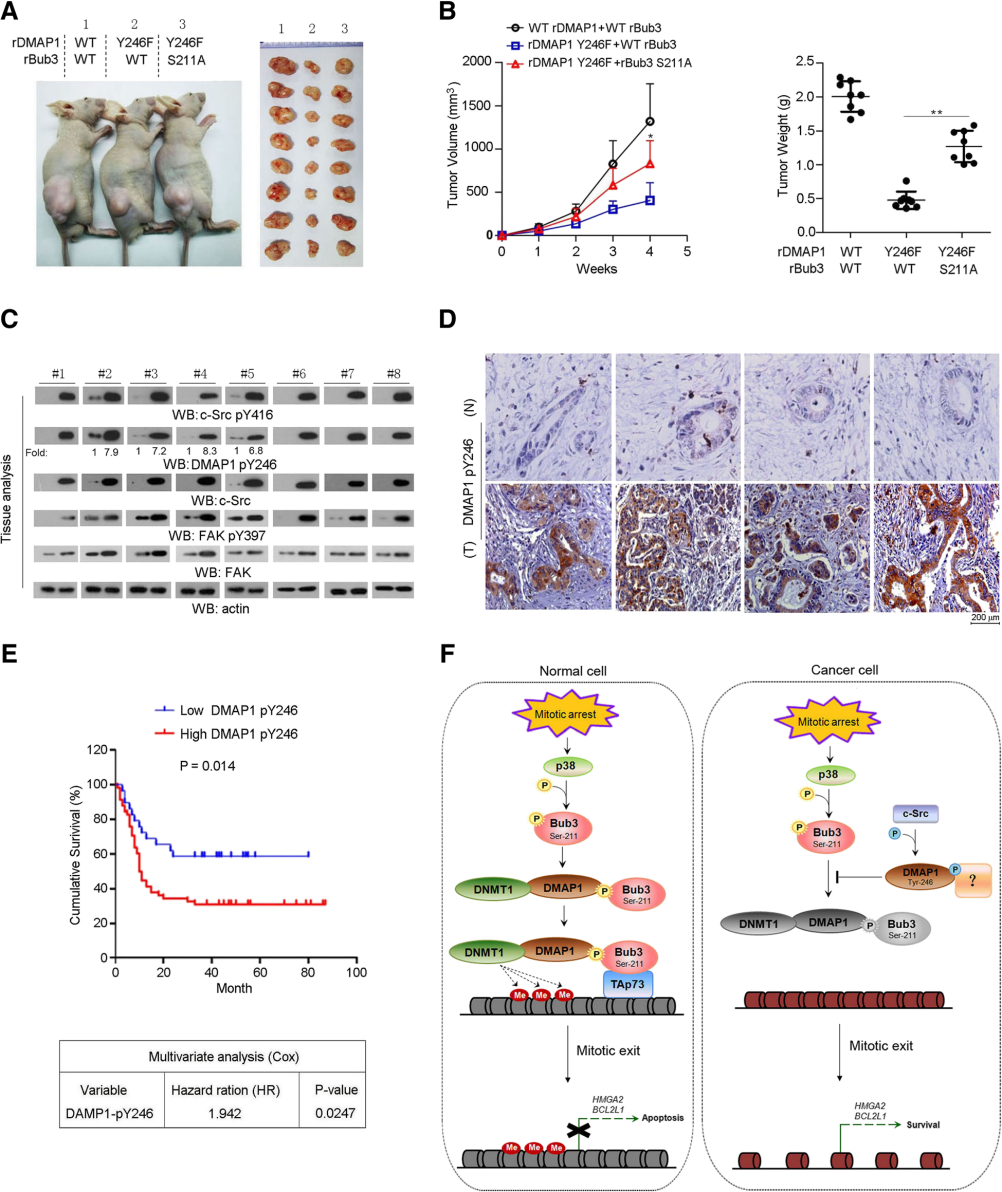
DMAP1 Y246 phosphorylation is required for tumourigenesis. **a** and **b**, A total of 5 × 10^6^ SW1990 cells with Bub3 or DMAP1 depletion and reconstituted expression of the WT rBub3/WT rDMAP1, WT rBub3/rDMAP1 Y246F or rBub3 S211A/rDMAP1 Y246F were subcutaneously injected into the athymic nude mice. Paclitaxel (5 mg/kg) was injected intraperitoneally every two days once the volume of tumours reached 200 mm^3^. Data represent the mean ± s.e.m. (*n* = 8). *represents *p* < 0.05 and **represents *p* < 0.01 between indicated groups. Representative tumour xenografts were shown (**a**). Tumour volumes and weight were measured. In the measurement of tumour volumes, length (a) and width (b) and calculated using the following equation: V = ab^2^/2. Data represent the means ± s.e.m. (*n* = 8, right panel) (**b**). **c** and **d** Immunoblotting analyses (**c**) and Immunohistochemical staining (**d**) with anti-DMAP1 pY246 was performed on human pancreas tumour specimens. Representative photos were shown. For immunoblotting analyses, the numbers underneath the blotting bands represent the normalized density quantified by using densitometry using Image J 2 × software. **e**, The survival times for 90 patients with low (0–2 staining scores, blue curve) versus high (2.1–5 staining scores, red curve) DMAP1 Tyr246 phosphorylation (low, 29 patients; high, 61 patients) (upper right) were compared. The Kaplan-Meier method and log-rank tests indicating the significance level of the association of DMAP1 Tyr246 phosphorylation (*p* = 0.014) with patient survival. The table (lower) shows the cox-multivariate analysis after adjustment for patient sex and age, indicating the significance level of the association of DMAP1 Tyr246 phosphorylation (*p* = 0.0247, HR = 1.942) with patient survival. **f**, The schematic diagram showing the regulatory role of Bub3/DMAP1 complex in DNA methylation upon mitotic stress in normal pancreatic epithelial cells and pancreatic cancer cells. Mitotic arrest induces p38 activation, which phosphorylates Bub3 at Ser 211 and promotes its interaction with DMAP1/DNMT1. TAp73 recruits Bub3/DMAP1 complex to promoter regions of its-targeted genes, where DMAP1/DNMT1 mediates DNA methylation and blocks expression of genes responsible for anti-apoptosis. c-Src can phosphorylate DMAP1 at Tyr246, which disrupts Bub3/DMAP1 complex formation. In normal cells with limited c-Src activity, p38/Bub3/DMAP1 signaling is readily activated under mitotic arrest; in turn, cells are prone to apoptosis (left part). In cancer cells, hyperactive c-Src leads to DMAP1 tyr246 phosphorylation and inhibits mitotic arrest-induced cell apoptosis mediated by p38/Bub3/DMAP1 signaling (right part)

c-Src-mediated DMAP1 Y246 phosphorylation promotes tumour cell survival through inhibition of Bub3/DMAP1 interaction under mitotic arrest (Fig. 4e). To further determine its implication in tumourigenesis, SW1990 cells expressed with WT rBub3/WT rDMAP1, WT rBub3/rDMAP1 Y246F and rBub3 S211A/rDMAP1 Y246F were subcutaneously injected into athymic nude mice, and Paclitaxel (5 mg/ kg) was injected intraperitoneally once the volume of tumours reached 200 mm^3^. Consequently, tumour cells with expression of WT rDMAP1 were still able to elicit rapid tumourigenesis with Paclitaxel treatment (Fig. 6a and b). In contrast, expression of rDMAP1 Y246F largely abrogated tumour growth. However, the suppressive effect of rDMAP1 Y246F was blocked by co-expression of rBub3 S211A. Consistently, IHC analyses of tumour tissues indicated rDMAP1 Y246F but not rDMAP1 Y246F/rBub3 S211A group exhibited much lower incidence of Ki67 staining and higher incidence of cleaved Caspase3 staining than WT counterparts (Additional file 6: Figure S6A). Meanwhile, real-time PCR analysis indicated expression of rDMAP1 Y246F but not rDMAP1 Y246F/rBub3 S211A led to a large increase of *BIRC3*, *BIRC5* and *NFKB1* mRNA levels in tumour tissues compared with WT counterparts (Additional file 6: Figure S6B), suggesting that *BIRC3*, *BIRC5*, *NFKB1*, whose function is relevant to inflammation, would be the critical downstream genes responsible for the anti-apoptotic-effects in vivo under the regulation by DMAP1/Bub3 complex. Collectively, these data indicated the inhibitory effect of DMAP1 Y246 phosphorylation on Bub3/DMAP1 interaction promoted tumour development.

### DMAP1 Y246 phosphorylation correlates prognosis in human tumour specimens

We demonstrated DMAP1 Y246 phosphorylation blocked Bub3-mediated transcriptional repression. To further define the clinical relevance of our finding, we first examined the c-Src activity and DMAP1 pY246 by immunoblotting of tissue extracts from 8 human pancreas tumour specimens. As a result, DMAP1 pY246 levels (Fig. 6c) were generally increased along with elevated c-Src/c-Src pY416 levels (Additional file 6: Figure S6C) in tumour tissues compared with the normal counterparts. In addition, IHC analyses were performed to examine DMAP1 pY246 levels in serial sections of 90 human pancreas tumour specimens (Fig. 6d). The specificity of antibody against DMAP1 Y246 phosphorylation was validated by using IHC analyses with specific blocking peptides (Additional file 6: Figure S6D). Survival duration of patients was evaluated with respect to low (0–2 staining) versus high (2.1–5 staining) level of DMAP1 Y246 phosphorylation (Fig. 6e and Additional file 6: Figure S6E). Patients whose tumours had low DMAP1 pY246 levels (29 cases) had a median survival that was not reached; those whose tumours had high DMAP1 pY246 levels (61 cases) had significantly lower median survival duration of 10.5 months. In a Cox multivariate model, the IHC scores of DMAP1 Y246 phosphorylation is an independent predictor of pancreatic cancer patient survival after adjusting for the age of the patient. These results reveal a tight relationship between DMAP1 Y246 phosphorylation and poor prognosis of human pancreatic cancer patient.

## Discussion

As the spindle checkpoint protein, Bub3 is responsible for the accurate chromosome segregation mainly through its inhibitory effect on APC/C-mediated ubiquitination prior to anaphase onset [2]. Besides spindle checkpoint components, Bub3 is also found to interact with proteins related to other cellular activities including DNA damage and gene transcription [27–29], revealing the multifaceted function of Bub3. In the present study, we found mitotic arrest induces Bub3 pS211 by p38, which leads to its binding to DMAP1. The association of Bub3 with TAp73 further promotes the recruitment of Bub3/DMAP1 complex to promoter of TAp73-targeted anti-apoptotic genes, where DMAP1 associates with DMNT1 and presumably eventually mediates DNA methylation that results in repression of gene reactivation following mitosis (Fig. 6f). In addition to its canonical function, these findings demonstrate a crucial role of Bub3 in the regulation of DNA methylation and subsequent gene transcription in response to mitotic stress. Particularly, our study indicates Bub3 S211 phosphorylation mediated by p38 activation is restricted on the condition of mitosis arrest but not TGF-β stimulation at interphase (Additional file 2: Figure S2F and 2G), which is consistent with the result that p38/Bub3 interaction is exclusively detected at mitosis (Fig. 2c). This suggests Bub3/DMAP1/DNMT1 axis is operated as a specific signaling feedback upon mitotic stress. Of note, although our data shows Bub3/TAp73 complex formation is responsible for initiation of DMAP1/Bub3-DNMT1 signaling, the mechanism of how Bub3 interacts with TAp73 remains to be further investigated.

Mitotic defect leads to growth arrest and the failure of its correction can cause cell death, which is considered as a negative selection to prevent the occurrence of aneuploidy [4, 5]. Frequently, cancer cells are able to escape from detrimental effects resulted by mitosis dysregulation and thus are prone to aneuploidy occurrence [41–43], revealing there exists according aberrant signals that enable cancer cells to counteract the relevant stress signals. In our study, we found pancreatic cancer cells display high level of DMAP1 pY246 mediated by c-Src; and this blocks mitotic arrest-induced Bub3/DMAP1 interaction and thus protects cancer cells from mitotic arrest-induced apoptosis. Hyperaction of c-Src has been importantly implicated in pancreatic cancer progression [39], the positive impacts of c-Src-mediated DMAP1 pY246 on tumour growth exemplifies that the adaption of cancer cells to mitotic defect-induced stress is addicted to strong c-Src activity. Moreover, since our study shows p38 activity and Bub3 pS211 are basally presented at a notable level in cancer cells, it could be assumed that adequate c-Src activity is required for cancer cells to counteract p38/Bub3/DMAP1-induced apoptosis not only under mitotic arrest induced artificially but also at routine process of mitosis progression. Indeed, this is supported by clinical analysis showing that the level of DMAP1 pY246 generally correlates to poor prognosis of pancreatic cancer patient. Regulation of epigenetic modifications at mitosis is linked to transcriptional reactivation in the subsequent cell cycle [20–22]. In consistence to this, our data indicates DMAP1 pY246 facilitates transcription of antiapoptotic-gene BCL2L1 under mitotic arrest in cancer cells, which is linked to its negative effects on Bub3 pS211-mediated DNA methylation at BCL2L1promoter (Fig. 5). As it cannot be excluded that DMAP1 pY246 would be involved in other cellular events in addition to DMAP1-Bub3 interaction, the precise effects of DMAP1 pY246 on Bub3-mediated DNA methylation needs to be further clarified.

The identification of the novel role of Bub3 in gene reactivation following mitotic arrest uncovers a flexible and instrumental insight into how epigenetic modifiers are constantly regulated in response to environmental signals. On the other hand, the inhibitory effect of c-Src on Bub3/DMAP1/DNMT1 axis illustrates a critical way for cancer cells to resist mitotic stress, and this finding importantly provides a molecular basis for improving the therapy of pancreatic cancer that are refractory to anti-mitosis treatment with upregulated c-Src activity.

## Conclusions

Our findings reveal Bub3/DMAP1 complex is crucial for responsive gene expression following mitotic arrest and demonstrate the survival advantage of cancer cells under mitotic stress is addicted to the inhibitory effect of c-Src on Bub3/DMAP1-mediated apoptosis.

## Additional files

Additional file 1: **Figure S1.** Bub3 interacts with DMAP1 during mitotic arrest. (DOCX 766 kb)

Additional file 2: **Figure S2.** p38 phosphorylates Bub3 at Ser211 and promotes Bub3/DMAP1 interaction. (DOCX 1430 kb)

Additional file 3: **Figure S3.** c-Src phosphorylates DMAP1 at Tyr246 and disrupts Bub3/DMAP1 complex formation. (DOCX 211 kb)

Additional file 4: **Figure S4.** Bub3/DMAP1 complex repressed anti-apoptotic genes transcription. (DOCX 1440 kb)

Additional file 5: **Figure S5.** c-Src-mediated DMAP1 phosphorylation blocks DMAP1-mediated DNA methylation. (DOCX 1090 kb)

Additional file 6: **Figure S6.** DMAP1 Y246 phosphorylation is required for Tumourigenesis. (DOCX 1290 kb)

## Abbreviations

APC/C: Anaphase-promoting complex/cyclosome
DMAP1: DNA methytransferase 1 associated protein
DNMT1: DNA methytransferase 1 (DNMT1)
HDACs: Histone deacetylases
HPDE: Human normal pancreatic duct epithelial
SAC: Spindle assembly checkpoint
