# Correction to: c-Src confers resistance to mitotic stress through inhibition of DMAP1/Bub3 complex formation in pancreatic cancer

**DOI:** 10.1186/s12943-019-1067-2

**Published:** 2019-11-29

**Authors:** Jingjie Li, Bin Hu, Ting Wang, Wenhua Huang, Chunmin Ma, Qin Zhao, Lingang Zhuo, Tao Zhang, Yuhui Jiang

**Affiliations:** 1 The Institute of Cell Metabolism, Shanghai Key Laboratory of Pancreatic Disease, Shanghai General Hospital, Shanghai Jiaotong University, School of Medicine, Shanghai, 200080 China; 2 Department of Gastroenterology, Shanghai General Hospital, Shanghai Jiao Tong University, School of Medicine, Shanghai, 200080 China; 3 Department of Pharmacology, College of Basic Medical Sciences, Tianjin Medical University, Tianjin, China; 4 Department of Orthopedics, Shanghai General Hospital, Shanghai Jiao Tong University, School of Medicine, Shanghai, 200080 China

**Correction to: Mol Cancer**

**https://doi.org/10.1186/s12943-018-0919-5**

Following publication of the work [1], authors reported the “flow cytometery plots” panel in Fig. 4e contained an inter-duplication in error. As shown in the original Fig. 4e, the “flow cytometery plots” image of “rDMAP1 Y246+Bcl-Xl” group was shown identical to “WT rDMAP1+WT rBub3” group. The authors confirm that the “flow cytometery plots” image of “rDMAP1 Y246+Bcl-Xl” group was mistakenly presented in the original Fig. 4, and that this mistake was resulted by unintentionally covering the correct image with the additional image in “WT rDMAP1+WT rBub3” group when during figure preparation, which is reflected by the repetition of the label (FL2-H) for the y axis appeared in the original image. The updated figure (Fig. 4e) is included in this correction. In addition, the authors noticed the word “of” was inadvertently omitted in the headline for this article. The corrected headline is suggested as “c-Src confers resistance to mitotic stress through inhibition of DMAP1/Bub3 complex formation in pancreatic cancer”.

The correction does not affect the findings or conclusions of the article. The authors apologize for any inconvenience that the inaccuracy may have caused.

The correct version of the figure is shown below.

**Fig. 4:**
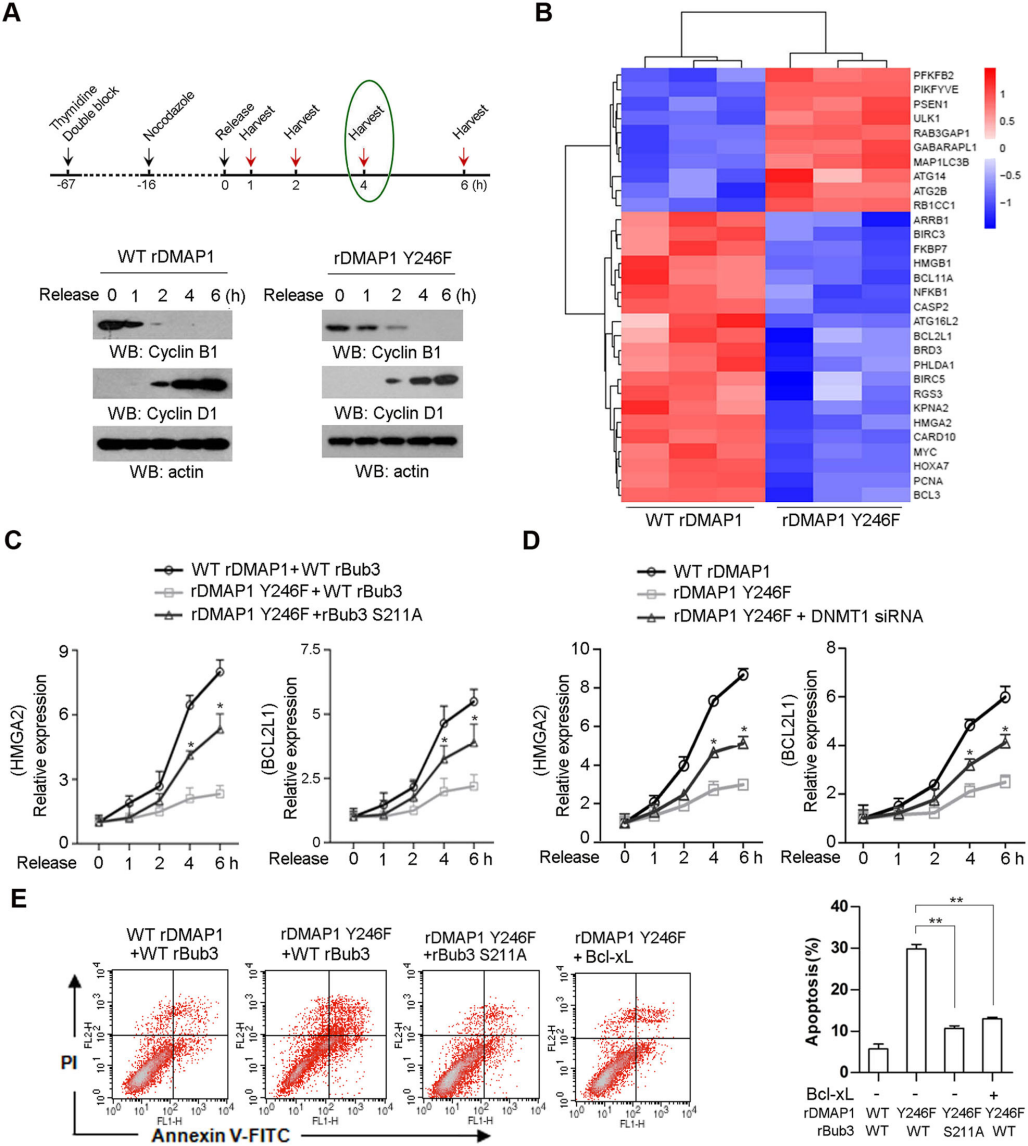
Bub3/DMAP1 complex represses anti-apoptotic genes transcription. In **a**, immunoblotting analyses were performed using the indicated antibodies; data represent 1 out of 3 experiments. In **c**-**e**, the values represent mean ± s.e.m. of three independent experiments. **a**, SW1990 cells were double blocked by thymide and treated with nocodazole (200 nM) following by releasing for the indicated periods. **b**, SW1990 cells were released for 4 h after thymidine double block and nocodazole (200 nM) for 16 h. Hierachical clustering of 4307 probe sets correlating with DMAP1 Y246F-expressed cells show that genes relevant to anti-apoptosis or autophagy were effective in separating cases from DMAP1 WT-expressed cells. **c** and **d** SW1990 cells expressed with the indicated plasmids were treated with nocodazole (200 nM) post thymidine double block, and were released for the indicated time. Relative mRNA levels were analyzed by real-time PCR. In **c**, * represents *p* < 0.05 between groups of cells expressing rDMAP1 Y246F plus WT rBub3 and groups of cells expressing rDMAP1 Y246F plus rBub3 S211A. In **d**, * represents *p* < 0.05 between groups of cells expressing rDMAP1 Y246F and groups of cells expressing rDMAP1 Y246F plus DNMT1 siRNA. **e**, Cell apoptosis was analyzed by Annexin V assays followed by flow cytometry. ** represents *p* < 0.01 between indicated groups
