# Supplementary material for: C-Src confers resistance to mitotic stress through inhibition DMAP1/Bub3 complex formation in pancreatic cancer
Source: Mol Cancer. 2018 Dec 15;17:174. doi: 10.1186/s12943-018-0919-5 (PMC6295060; doi:10.1186/s12943-018-0919-5)
Supplement: Supplementary file 1 — Figure S1. Bub3 interacts with DMAP1 during mitotic arrest. (DOCX 766 kb) [file 12943_2018_919_MOESM1_ESM.docx]

**Additional file 1**

**Figure S1. Bub3 interacts with DMAP1 during mitotic arrest.**

(A) HPDE cells expressing Flag-Bub3 were synchronized in interphase by thymidine double block (2 mM) or were synchronized in mitosis by nocodazole (200 nM) treatment for 16 h after releasing thymidine double block for 8 h (left panel). Cellular extracts subjected to immunoprecipitation with an anti-Flag antibody were analyzed by mass spectrometry. Bub3-interacting proteins identified by mass spectrometry were shown. (B) HPDE cells synchronized by thymidine double block (2 mM) were released for 8 h, followed by nocodazole (200 nM) treatment for 16 h (left panel), and cellular extracts were subjected to immunoprecipitation with an anti-Bub3 antibody. Immunoblotting analyses were performed using the indicated antibodies. (C) HPDE cells synchronized by thymidine double block (2 mM) were released for 8 h, followed by nocodazole (200 nM) treatment for 16 h (left panel), and cellular extracts were subjected to immunoprecipitation with an anti-Bub3 antibody. Immunoblotting analyses were performed using the indicated antibodies. (D) HPDE cells synchronized by thymidine double block (2 mM) were released for 8 h, followed by nocodazole (200 nM) treatment for 16 h and release for 6 h, and cellular extracts collected at indicated time points were subjected to immunoprecipitation with an anti-Bub3 antibody. Immunoblotting analyses were performed using the indicated antibodies. (E) HPDE cells synchronized by thymidine double block (2 mM) were released for 8 h, followed by nocodazole (200 nM) treatment for 16 h (left panel), and cellular extracts were subjected to immunoprecipitation with an anti-DMAP1 antibody. Immunoblotting analyses were performed using the indicated antibodies. (F) HPDE cells were transfected with or without DNMT1 siRNA duplex. Cellular extracts were subjected to immunoprecipitation with an anti-Bub3 antibody. (G) PANC-1 and SW1990 cells expressing Flag-Bub3 were synchronized in interphase by thymidine double block (2 mM) or were synchronized in mitosis by nocodazole (200 nM) treatment for 16 h after releasing thymidine double block for 8 h (left panel). The efficiency of synchronization was examined by immunoblotting analyses of H3 pS10 or flow cytometry analyses. (H) PANC-1 cells were synchronized by thymidine double block (2 mM) and were released for 8 h, followed by nocodazole (200 nM) treatment for 16 h (left panel). PANC-1 cells were treated with SU6656 (shown as ‘SU’) (10 μM) for indicated length of time post nocodazole treatment for 16 h. Cellular extracts were subjected to immunoblotting analyses with indicated antibodies.
