# Supplementary material for: C-Src confers resistance to mitotic stress through inhibition DMAP1/Bub3 complex formation in pancreatic cancer
Source: Mol Cancer. 2018 Dec 15;17:174. doi: 10.1186/s12943-018-0919-5 (PMC6295060; doi:10.1186/s12943-018-0919-5)
Supplement: Supplementary file 2 — Figure S2. p38 phosphorylates Bub3 at Ser211 and promotes Bub3/DMAP1 interaction. (DOCX 1430 kb) [file 12943_2018_919_MOESM2_ESM.docx]

**Additional file 2**

**Figure S2. p38 phosphorylates Bub3 at Ser211 and promotes Bub3/DMAP1 interaction.**

(A) HPDE cells were synchronized in interphase (I) by thymidine double block (2 mM) or were synchronized in mitosis (M) by nocodazole (200 nM) treatment for 16 h after releasing thymidine double block for 8 h. Cellular extracts were subjected to immunoprecipitation with an anti-Bub3 antibody and the immunoprecipitates were treated with or without CIP (10 units) and CIP/Na_3_VO_4_. Immunoblotting analyses were performed using the indicated antibodies. (B) HPDE cells were synchronized in interphase (I) by thymidine double block (2 mM) or were synchronized in mitosis (M) by nocodazole (200 nM) treatment for for 6 h (shown as ‘S’) or for 16 h (shown as ‘L’) after releasing thymidine double block for 8 h. Immunoblotting analyses were performed using the indicated antibodies. (C) HPDE cells were synchronized in interphase (I) by thymidine double block (2 mM) or were synchronized in mitosis (M) by nocodazole (200 nM) treatment for 16 h after releasing thymidine double block for 8 h. Cells were treated with Compound C (10 μM), SP600125 (20 μM) and SB203580 (25 μM) for 1 h, after Nocodazole treatment for 6 h. Immunoblotting analyses were performed using the indicated antibodies. (D) HPDE cells were synchronized in interphase (I) by thymidine double block (2 mM) or were synchronized in mitosis (M) by nocodazole (200 nM) treatment for 16 h after releasing thymidine double block for 8 h; then cells were treated with SB203580 (10 μM) for 1 h post nocodazole treatment for 6 h. Immunoblotting analyses were performed using the indicated antibodies. (E) HPDE cells transfected with p38 siRNA were synchronized in interphase (I) by thymidine double block (2 mM) or were synchronized in mitosis (M) by nocodazole (200 nM) treatment for 16 h after releasing thymidine double block for 8 h. Immunoblotting analyses were performed using the indicated antibodies. (F) HPDE cells were synchronized in interphase (I) by thymidine double block (2 mM) or were synchronized in mitosis (M) by nocodazole (200 nM) treatment for 16 h after releasing thymidine double block for 8 h; then cells were treated with RO3306 (10 μM) for 4 h post nocodazole treatment for 6 h. Immunoblotting analyses were performed using the indicated antibodies. (G) HPDE cells synchronized in interphase (I) by thymidine double block (2 mM) were treated with or without TGF-β for 1 h. Immunoblotting analyses were performed using the indicated antibodies. (H) The indicated purified His-Bub3 protein was mixed with GST-DMAP1 purified protein with or without p38. GST pull down analyses were performed. (I) HPDE cells were expressed with a vector for Bub3 shRNA, and reconstituted with expression of WT rBub3 or indicated rBub3 mutants. Immunoblotting analyses were performed using the indicated antibodies. (J) PANC-1 expressed with indicated Flag-Bub3 were synchronized in interphase (I) by thymidine double block (2 mM) or were synchronized in mitosis (M) by nocodazole (200 nM) treatment for 16 h after releasing thymidine double block for 8 h. Cells were treated with or without SU6656 (shown as ‘SU’) (10 μM) for 1 h post nocodazole treatment for 10 h. Cellular extracts were subjected to immunoprecipitation with an anti-Flag antibody. Immunoblotting analyses were performed using the indicated antibodies. (K) HPDE cells synchronized in mitosis were expressed with indicated Flag-Bub3. Cellular extracts were subjected to immunoprecipitation with an anti-Flag antibody. Immunoblotting analyses were performed using the indicated antibodies.
