# Supplementary material for: C-Src confers resistance to mitotic stress through inhibition DMAP1/Bub3 complex formation in pancreatic cancer
Source: Mol Cancer. 2018 Dec 15;17:174. doi: 10.1186/s12943-018-0919-5 (PMC6295060; doi:10.1186/s12943-018-0919-5)
Supplement: Supplementary file 3 — Figure S3. c-Src phosphorylates DMAP1 at Tyr246 and disrupts Bub3/DMAP1 complex formation. (DOCX 211 kb) [file 12943_2018_919_MOESM3_ESM.docx]

**Additional file 3**

**Figure S3. c-Src phosphorylates DMAP1 at Tyr246 and disrupts Bub3/DMAP1 complex formation.** (A) PANC-1 expressed with indicated Flag-Bub3 were synchronized by thymidine double block (2 mM) and were released for 8 h, followed by nocodazole (200 nM) treatment for 16 h. Cells were treated with or without SU6656 (shown as ‘SU’) (10 μM) for 1 h post nocodazole treatment for 10 h. Immunoblotting analyses were performed using the indicated antibodies. (B) PANC-1 cells were synchronized in interphase or mitosis. Cellular extracts were subjected to immunoprecipitation with an anti-Src antibody. Immunoblotting analyses were performed using the indicated antibodies.
