# Supplementary material for: C-Src confers resistance to mitotic stress through inhibition DMAP1/Bub3 complex formation in pancreatic cancer
Source: Mol Cancer. 2018 Dec 15;17:174. doi: 10.1186/s12943-018-0919-5 (PMC6295060; doi:10.1186/s12943-018-0919-5)
Supplement: Supplementary file 4 — Figure S4. Bub3/DMAP1 complex repressed anti-apoptotic genes transcription. (DOCX 1440 kb) [file 12943_2018_919_MOESM4_ESM.docx]

**Additional file 4**

**Figure S4. Bub3/DMAP1 complex repressed anti-apoptotic genes transcription.**

In B, D-F, the values represent mean ± s.e.m. of three independent experiments. (A) SW1990 cells were expressed with a vector for control shRNA or DMAP1 shRNA and reconstituted with expression of WT rDMAP1 or rDMAP1 Y246F. Immunoblotting analyses were performed using the indicated antibodies; (B) SW1990 cells were expressed with a vector for control shRNA or DMAP1 shRNA and reconstituted with expression of WT rDMAP1 or rDMAP1 Y246F. Cells were unsynchronized (left panel), or double blocked by thymide and treated with nocodazole (200 nM) (middle panel) or nocodazole (200 nM) following by releasing for 6 h (right panel). Flow cytometry analyses were performed. (C) SW1990 cells were expressed with a vector for control shRNA or DMAP1 shRNA and Bub3 shRNA, and reconstituted with expression of WT rDMAP1 or rDMAP1 Y246F and WT rBub3 or rBub3 S211A. Immunoblotting analyses were performed using the indicated antibodies. (D) SW1990 cells expressed with the indicated plasmids were treated with nocodazole (200 nM) post thymidine double block, and were released for the indicated time. Relative mRNA levels were analyzed by realtime PCR. *represents p<0.05 between groups of cells expressing rDMAP1 Y246F plus WT rBub3 and groups of cells expressing rDMAP1 Y246F plus rBub3 S211A. (E) SW1990 cells expressed with the indicated plasmids were treated with nocodazole (200 nM) post thymidine double block, and were released for the indicated time. Relative mRNA levels were analyzed by realtime-PCR. *represents p<0.05 between groups of cells expressing rDMAP1 Y246F and groups of cells expressing rDMAP1 Y246F plus DNMT1 siRNA. (F) Cell apoptosis was analyzed by Annexin V assays followed by flow cytometry. *represents p<0.05 between indicated groups, ** represents p<0.01 between indicated groups.
