# Supplementary material for: C-Src confers resistance to mitotic stress through inhibition DMAP1/Bub3 complex formation in pancreatic cancer
Source: Mol Cancer. 2018 Dec 15;17:174. doi: 10.1186/s12943-018-0919-5 (PMC6295060; doi:10.1186/s12943-018-0919-5)
Supplement: Supplementary file 5 — Figure S5. c-Src-mediated DMAP1 phosphorylation blocks DMAP1-mediated DNA methylation. (DOCX 1090 kb) [file 12943_2018_919_MOESM5_ESM.docx]

**Additional file 5**

**Figure S5. c-Src-mediated DMAP1 phosphorylation blocks DMAP1-mediated DNA methylation.**

(A-D), SW1990 cells were reconstituted expressed with WT rBub3+ WT rDMAP1, rBub3WT+rDMAP1Y246F and rBub3S211A+rDMAP1Y246F as mentioned in Fig. S4 C; cells expressed with the indicated plasmids were synchronized in mitosis (M) by nocodazole (200 nM) treatment for 16 h after releasing thymidine double block for 8 h. The values represent from 2 repeated samples. (A), Circos plots representation of genome-wide DNA methylation levels. The outermost tracks represent the 23 chromosomes in the genome. The other tracks represent the following: 1, gene density. Red, plus-strand gene; green,minus-strand gene; 2, Average levels for all the CGs in 10-Mbp-wide windows; 3, CG differentially methylated regions (DMRs). (B), Percentages of mCs in each context. (C), DNA methylation levels of centromeric regions of all 22 autosomes and­ the X chromosome were presented as ratio of methylated reads to unmethylated reads. (D), DNA methylation level of genomic features displayed as ratio of methylated reads to unmethylated reads. (E) SW1990 (left panel) and PANC-1(right panel) cells expressed with the indicated plasmids were synchronized in mitosis (M) by nocodazole (200 nM) treatment for 16 h after releasing thymidine double block for 8 h. 5-mc levels were examined by bisulfite sequencing using primers covering TAp73 binding site of BCL2L1 gene promoter region. (F) SW1990 cells expressed with the indicated plasmids were synchronized in interphase (I) by thymidine double block (2 mM) or were synchronized in mitosis (M) by nocodazole (200 nM) treatment for 16 h after releasing thymidine double block for 8 h. Cellular extracts were subjected to immunoprecipitation with an anti-Flag antibody. (G) SW1990 cells were synchronized in interphase (I) by thymidine double block (2 mM) or were synchronized in mitosis (M) by nocodazole (200 nM) treatment for 16 h after releasing thymidine double block for 8 h. ChIP analyses were performed using an anti-Bub3 antibody. The primers covering a specific region of chromosome 12 or TAp73 binding site of *BCL2L1* gene promoter region (right panel) were used for the real-time PCR. The y axis shows the value normalized to the input. Data represent mean ± s.e.m. of three independent experiments; **represents p<0.01 between indicated groups. (H, I) SW1990 cells expressed with the indicated plasmids were synchronized in interphase (I) by thymidine double block (2 mM) or were synchronized in mitosis (M) by nocodazole (200 nM) treatment for 16 h after releasing thymidine double block for 8 h. ChIP analyses were performed using an anti-DNMT1 antibody (H) or anti-DMAP1 pY246 antibody (I). The primers covering TAp73 binding site of *BCL2L1* gene promoter region were used for the real-time PCR. The y axis shows the value normalized to the input. The values represent mean ± s.e.m. of three independent experiments; **represents p<0.01 between indicated groups.­­­
