# Supplementary material for: C-Src confers resistance to mitotic stress through inhibition DMAP1/Bub3 complex formation in pancreatic cancer
Source: Mol Cancer. 2018 Dec 15;17:174. doi: 10.1186/s12943-018-0919-5 (PMC6295060; doi:10.1186/s12943-018-0919-5)
Supplement: Supplementary file 6 — Figure S6. DMAP1 Y246 phosphorylation is required for Tumourigenesis. (DOCX 1290 kb) [file 12943_2018_919_MOESM6_ESM.docx]

**Additional file 6**

**Figure S6. DMAP1 Y246 phosphorylation is required for Tumourigenesis.** In A-C, and E, data represent the mean ± s.e.m. (n=8). *represents p<0.05 and **represents p<0.01 between indicated groups. (A) Immunohistochemical staining with anti-Ki67 or cleaved caspase3 was performed on xenograft tumour tissues. Representative photos were shown (left panel). (B) A total of 5×10^6^ SW1990 cells with Bub3 or DMAP1 depletion and reconstituted expression of the WT rBub3/WT rDMAP1, WT rBub3/rDMAP1 Y246F or rBub3 S211A/rDMAP1 Y246F were subcutaneously injected into the athymic nude mice. Paclitaxel (5 mg/kg) was injected intraperitoneally every two days once the volume of tumours reached 200 mm^3^. The extracts from xenograft tumour tissues were used for the real-time PCR. (C) Human pancreatic tumour specimens were used for the real-time PCR. (D) Immunohistochemical staining of human pancreatic tumour specimens were performed with the indicated antibodies in the presence or absence of specific blocking peptides. (E) Immunohistochemical staining of human pancreatic tumour specimens were perfomed with the antibody against DMAP1pY246. DMAP1 pY246 levels were quantified by using Image-Pro Plus 6.0 software and were shown as the density mean (integrated option density (IOD)/area).
